# Transcriptomic analysis reveals a global alkyl-quinolone-independent regulatory role for PqsE in facilitating the environmental adaptation of *Pseudomonas aeruginosa* to plant and animal hosts

**DOI:** 10.1111/j.1462-2920.2010.02214.x

**Published:** 2010-06

**Authors:** Giordano Rampioni, Christian Pustelny, Matthew P Fletcher, Victoria J Wright, Mary Bruce, Kendra P Rumbaugh, Stephan Heeb, Miguel Cámara, Paul Williams

**Affiliations:** 1 School of Molecular Medical Sciences, Centre for Biomolecular Sciences, University Park, University of NottinghamNottingham NG7 2RD, UK; 2 Department of Surgery, Texas Tech University, Health Sciences CenterLubbock, TX 79430, USA

## Abstract

The quorum sensing (QS) system of *Pseudomonas aeruginosa* constitutes a sophisticated genome-wide gene regulatory network employing both *N*-acylhomoserine lactone and 2-alkyl-4-quinolone (AQ) signal molecules. AQ signalling utilizes 2-heptyl-3-hydroxy-4-quinolone (PQS) and its immediate precursor, 2-heptyl-4-quinolone (HHQ). AQ biosynthesis requires the first four genes of the *pqsABCDE* operon and while the biochemical function of *pqsE* is not known, it is required for the production of secondary metabolites such as pyocyanin. To gain insights into the relationship between the AQ stimulon, the PqsE stimulon and the regulatory function of PqsE, we constructed a *pqsE* inducible mutant (*pqsE*ind) and compared the transcriptomes of the induced and uninduced states with a *pqsA* mutant. Of 158 genes exhibiting altered expression in the *pqsA* mutant, 51% were also affected in the *pqsE* mutant. Following induction of *pqsE*, 237 genes were differentially expressed compared with the wild-type strain. In the *pqsE*ind strain, *pqsA* was highly expressed but following induction both *pqsA* expression and AQ biosynthesis were repressed, revealing a negative autoregulatory role for PqsE. Furthermore, *pqsE* was required for swarming motility and virulence in plant and animal infection models in the absence of AQs, while mature biofilm development required both *pqsA* and *pqsE*. Taken together these data reveal that PqsE is a key regulator within the QS circuitry facilitating the environmental adaptation of *P. aeruginosa*.

## Introduction

*Pseudomonas aeruginosa* is an opportunistic pathogen capable of infecting humans, animals, plants and insects and causing both acute and chronic infections. It produces a wide variety of secondary metabolites and virulence determinants, readily forms biofilms and is naturally resistant to many antimicrobial agents. Many of the structural genes involved are under quorum sensing (QS) control and are regulated in a population density-dependent manner via both *N*-acylhomoserine lactone (AHL) and 2-alkyl-4-quinolone (AQ) signalling pathways. These comprise the AHL-dependent hierarchically linked *las* and *rhl* systems which are inter-connected with a third QS system that utilizes AQ signal molecules (recently reviewed by Williams and Cámara, 2009). Although *P. aeruginosa* produces over 50 different AQ congeners, the two major AQs which function as QS signals are 2-heptyl-3-hydroxy-4-quinolone (the ‘*Pseudomonas*Quinolone Signal’; PQS) and its immediate precursor 2-heptyl-4-quinolone (HHQ) (Pesci *et al*., 1999; Déziel *et al*., 2004; Diggle *et al*., 2007). Multiple genes are required for AQ biosynthesis and signal transduction. These are mostly arranged in an operon, *pqsABCDE*, which is under the positive control of the transcriptional regulator, PqsR(MvfR) (Cao *et al*., 2001; Gallagher *et al*., 2002). The first four gene products of this operon are required for HHQ biosynthesis. HHQ is oxidized to PQS via the action of the putative monooxygenase PqsH, coded by the *pqsH* gene located some distance downstream of the *pqsABCDE* operon (Gallagher *et al*., 2002; Déziel *et al*., 2004).

The biosynthesis of AQs occurs via the ‘head-to-head’ condensation of anthranilate (supplied via *phnAB* operon or the *kynABU* genes) and a 3-oxo-fatty acid (Ritter and Luckner, 1971; Bredenbruch *et al*., 2005). Anthranilate is primed by PqsA for entry into the HHQ biosynthetic pathway (Coleman *et al*., 2008), whereas PqsD acts as a condensing enzyme which may either catalyse thecondensation of anthranoyl-CoA with a 3-oxo-acid, or may be involved in the formation of a 3-oxo-acid precursor (Zhang *et al*., 2008). PqsB and PqsC are highly homologous to 3-oxoacyl-(acyl-carrier-protein) synthases and while their precise contribution to AQ biosynthesis is not yet understood, they are likely to be involved in fatty acid recruitment and condensation (Gallagher *et al*., 2002).

A positive feedback loop exists within the AQ signalling pathway, because both PQS and HHQ can bind to PqsR and drive the expression of the *pqsA* promoter so enhancing *pqsABCDE* expression (McGrath *et al*., 2004; Wade *et al*., 2005; Xiao *et al*., 2006a,b; Diggle *et al*., 2007). AQ signalling is linked to AHL-dependent QS because the *las* system exerts positive regulatory control upon *pqsR* and *pqsH* (Déziel *et al*., 2004) while *rhl* negatively impacts on *pqsABCDE* and *pqsR* expression (McGrath *et al*., 2004; Wade *et al*., 2005; Xiao *et al*., 2006a). Exoproducts such as the rhamnolipids and elastase are synergistically regulated by AHL- and AQ-dependent QS (McKnight *et al*., 2000; Farrow *et al*., 2008). Furthermore, LasR has recently been shown to bind to the promoters of both *pqsR* and *pqsA in vivo* (Gilbert *et al*., 2009).

The fifth gene in the *pqs* operon, *pqsE*, is not required for AQ biosynthesis but instead is an effector of the AQ response (Gallagher *et al*., 2002; Diggle *et al*., 2003; Déziel *et al*., 2004; Farrow *et al*., 2008). At present the precise function of PqsE is not understood. Sequence and structural analyses indicate that PqsE belongs to the family of enzymes known as metallo-β-hydrolases, because it possesses the characteristic amino acid ‘HXHXDH’ motif of these proteins and has a metallo-β-lactamase fold with an Fe^2+^/Fe^3+^ centre in the active site (although this may not be the true cofactor) (Yu *et al*., 2009). Mutation of *pqsE* results in the reduced production of PQS-mediated exoproducts such as pyocyanin, elastase and rhamnolipid, even though the PQS and AHL levels are similar to wild-type (Gallagher *et al*., 2002; Diggle *et al*., 2003; Déziel *et al*., 2005; Farrow *et al*., 2008). PqsE was therefore termed the ‘PQS signal response’ protein. However, overexpression of PqsE enhanced pyocyanin, elastase and rhamnolipid production in the absence of PQS and PqsR (Farrow *et al*., 2008). Such regulation was *rhl*-dependent and PqsE was also shown to enhance the ability of *E. coli* expressing *rhlR* to respond to *N*-butanoylhomoserine lactone (C4-HSL) with respect to *rhlA* expression (Farrow *et al*., 2008).

The AQ-independent action of PqsE suggests that the primary function of PqsR in regulating the expression of key target genes is to drive the expression of *pqsE*. However, this does not explain why exogenous PQS but not HHQ can fully restore pyocyanin and Lectin A production in a *P. aeruginosa* PAO1 *pqsA* mutant, because both AQs drive the expression of *pqsA* via PqsR (Xiao *et al*., 2006a; Diggle *et al*., 2007). Given that PQS, in addition to functioning as a QS signal molecule, is an iron chelator (Bredenbruch *et al*., 2006; Diggle *et al*., 2007), a pro-oxidant and an inducer of an anti-oxidative stress response (Häussler and Becker, 2008), required for biofilm maturation (D’argenio *et al*., 2002; Allesen-Holm *et al*., 2006) and vesicle formation (Mashburn and Whiteley, 2005; Mashburn-Warren *et al*., 2008), it is probably involved in the regulation of both PqsE-dependent and PqsE-independent genes. Consequently, PqsE is likely to be responsible for regulating a subset of the genes controlled by the AQs. Therefore, to gain better insights into the relationship between the AQ stimulon, the PqsE stimulon and the regulatory function of PqsE, we first constructed an isopropyl β-d-l-thiogalactopyranoside (IPTG)-inducible *pqsE* mutant (*pqsE*ind) to facilitate control of *pqsE* expression independent of the *pqsA* promoter. By comparing the transcriptomes of the *pqsEind* mutant in the absence or presence of IPTG with that of a wild-type and a *pqsA* mutant strain we were able to define the nature of the AQ and PqsE stimulons and their inter-relationship. Furthermore, we show that PqsE (i) is required for swarming motility and biofilm development, and (ii) restores virulence in nematode, plant and mouse infection models in a *pqsA* mutant strain (i.e. in the absence of AQs). PqsE was also found to control negatively its own expression through the repression of *pqsA* transcription, so providing an additional layer of fine-tuning regulatory sophistication to QS in *P. aeruginosa*.

## Results

### Characterization of the *pqsA* stimulon

To identify genes regulated by the AQs, we used high-density oligonucleotide genomic microarrays to compare the transcriptional profiles of *P. aeruginosa* PAO1 and an isogenic *pqsA* mutant which does not produce AQs (Diggle *et al*., 2006; Fletcher *et al*., 2007). Under the experimental conditions employed, the growth curves for the parent and *pqsA* mutant were virtually identical (data not shown). RNA for transcriptomic analysis was extracted from cells grown to an optical density at 600 nm (OD_600_) of 1.5, corresponding to the late exponential phase of growth. This was because reverse-transcriptase polymerase chain reaction (RT-PCR) analysis showed that transcripts for all of the genes in the *pqsABCDE* operon reach a maximum level of expression at this stage of growth (Appendix S1 and Fig. S1A). The results of the microarray analysis revealed that the expression of 158 genes (2.8% of all *P. aeruginosa* genes) is affected by the *pqsA* mutation at this stage of growth (full results are given in Appendix S1 and Table S1). A significant number (31%) of these have been identified previously as genes controlled by QS via the AHLs (27%; Appendix S1 and Table S1; Hentzer *et al*., 2003; Schuster *et al*., 2003; Wagner *et al*., 2003) or involving components of the AQ-dependent QS system (21.5%; Déziel *et al*., 2004; Bredenbruch *et al*., 2006). Considering the hierarchical organization of the QS systems of *P. aeruginosa* (Williams and Cámara, 2009) an overlap between the transcripts identified in these experiments and those previously reported to be controlled by the *las* and *rhl* systems was anticipated. However, our finding that many of the genes modulated by the *pqsA* mutation were absent from previous analyses, highlights the complexity of the reciprocal regulation occurring within the *P. aeruginosa* QS network (Duan and Surette, 2007; Dekimpe and Déziel, 2009).

Among the 104 genes downregulated in the *pqsA* mutant (Table 1 and Table S1) we found genes involved in the production of virulence determinants, such as exoproteases (*aprX*, *aprD*), the ChiC extracellular chitinolytic enzyme (*chiC*), the LecA lectin (*lecA*), and secondary metabolites such as pyocyanin (*phzA1*, *phzC1*, *phzE1*, *phzA2*, *phzE2*, *phzC2*), PA2274 (a putative flavin-dependent monooxygenase), genes involved in pyochelin biosynthesis, uptake and regulation (*pchA*, *pchB*, *pchC*, *pchD*, *pchE*, *pchF*, *pchI*, *pchR*, *ftpA*), pyoverdine biosynthesis (PA2412 and *pvdH*), in antibiotic resistance (*mexG*, *mexH*, *mexI*, *opmD*), and in biofilm development (*tadA*, *tadZ*) (Duong *et al*., 1992; 2001; Folders *et al*., 2001; Mavrodi *et al*., 2001; Ravel and Cornelis, 2003; Aendekerk *et al*., 2005; Diggle *et al*., 2006; Tomich *et al*., 2007). Many of the genes identified above have also been previously identified in microarray experiments focusing on PQS signalling via characterization of the PqsR (MvfR) regulon in *P. aeruginosa* PA14 (Déziel *et al*., 2004) and the response of the *P. aeruginosa* PAO1 wild type to exogenously supplied PQS (Bredenbruch *et al*., 2006). Similarly, we have previously reported that *pqsA*, *lecA* and the pyochelin genes are downregulated in a *pqsA* mutant (Diggle *et al*., 2007). These findings validate the array data presented here.

**Table 1:** List of selected genes identified in the microarray analyses.

PA number^a^	Gene name^a^	wt vs *pqsA*^b^	wt vs *pqsE*ind^c^	wt vs *pqsE*ind + IPTG^d^	Product name^a^
PA0051	*phzH*			−1.597	Potential phenazine-modifying enzyme
PA0236 TR	*–*	1.538			Probable transcriptional regulator
PA0996 *	*pqsA*	12.420		2.897	Probable coenzyme A ligase
PA0997 ***	*pqsB*	7.350		3.801	Homologous to beta-keto-acyl-acyl-carrier protein synthase
PA0998 *	*pqsC*	6.597		3.735	Homologous to beta-keto-acyl-acyl-carrier protein synthase
PA0999 *	*pqsD*	6.466		2.804	3-Oxoacyl-[acyl-carrier-protein] synthase III
PA1000 *	*pqsE*	3.418			Quinolone signal response protein
PA1001 *	*phnA*	10.187			Anthranilate synthase component I
PA1002 *	*phnB*	3.462			Anthranilate synthase component II
PA1100	*fliE*			−1.570	Flagellar hook-basal body complex protein FliE
PA1104	*fliI*			−1.561	Flagellum-specific ATP synthase FliI
**PA1196 TR**	*–*	−1.516			Probable transcriptional regulator
PA1245 **	*aprX*	1.515			Hypothetical protein
**PA1246**	*aprD*	1.624			Alkaline protease secretion protein AprD
PA1452	*flhA*			−1.509	Flagellar biosynthesis protein FlhA
PA1603 TR	*–*	−1.590			Probable transcriptional regulator
PA1899	*phzA2*	2.556			Probable phenazine biosynthesis protein
**PA1901**	*phzC2*	2.233			Phenazine biosynthesis protein PhzC
**PA1903**	*phzE2*	2.016			Phenazine biosynthesis protein PhzE
PA2128	*cupA1*	−2.062	−1.581		Fimbrial subunit CupA1
**PA2147**	*katE*			−1.925	Catalase HPII
PA2273 TR	*soxR*	2.947	2.099		Probable transcriptional regulator
PA2274 ***	*–*	17.122	3.987		Hypothetical protein
PA2300 *	*chiC*	1.707			Chitinase
PA2413	*pvdH*	1.674			L-2,4-diaminobutyrate:2-ketoglutarate 4-aminotransferase
PA2507	*catA*	−3.125		−5.165	Catechol 1,2-dioxygenase
PA2508	*catC*	−2.544	4.185	−3.756	Muconolactone delta-isomerase
PA2509	*catB*	−3.597	3.577	−6.172	Muconate cycloisomerase I
PA2519 TR	*xylS*	−2.077			Transcriptional regulator XylS
PA2570 *	*lecA*	2.219			LecA
PA3095	*xcpZ*			−1.895	General secretion pathway protein M
PA3096	*xcpY*			−1.620	General secretion pathway protein L
PA3098	*xcpW*			−1.813	General secretion pathway protein J
PA3099	*xcpV*			−1.948	General secretion pathway protein I
PA3102	*xcpS*			−1.520	General secretion pathway protein F
PA3718	*-*	2.242			Probable MFS transporter
**PA3875**	*narG*	−2.552			Respiratory nitrate reductase alpha chain
PA3879	*narL*	−2.216			Two-component response regulator NarL
PA4205 ***	*mexG*	2.944			Hypothetical protein
PA4206 ***	*mexH*	5.338			RND efflux membrane fusion protein precursor
PA4207 *	*mexI*	7.925	1.886		RND efflux transporter
PA4208 *	*opmD*	20.813	6.835		Probable outer membrane protein precursor
**PA4210**	*phzA1*	2.440	1.519		Probable phenazine biosynthesis protein
PA4212 *	*phzC1*	2.131			Phenazine biosynthesis protein PhzC
PA4214 *	*phzE1*	1.678		−1.629	Phenazine biosynthesis protein PhzE
PA4221 **	*fptA*	7.076	5.448	6.577	Fe(III)-pyochelin outer membrane receptor precursor
PA4222 **	*pchI*	7.157	11.923	7.876	Probable ATP-binding component of ABC transporter
PA4225 **	*pchF*	71.483	57.723	75.785	Pyochelin synthetase
PA4226 **	*pchE*	139.217	94.197	124.376	Dihydroaeruginoic acid synthetase
PA4227 **^TR^	*pchR*	8.728	3.245	7.757	Transcriptional regulator PchR
PA4228 **	*pchD*	21.066	16.892	20.643	Pyochelin biosynthesis protein PchD
PA4229 **	*pchC*	1.611			pyochelin biosynthetic protein PchC
PA4230 **	*pchB*	27.171	15.570		Salicylate biosynthesis protein PchB
PA4231 **	*pchA*	32.538	21.940	38.831	Salicylate biosynthesis isochorismate synthase
**PA4300**	*tadC*			−1.525	TadC
**PA4302**	*tadA*	1.738			TadA ATPase
**PA4303**	*tadZ*	1.946			TadZ
PA4613 **	*katB*			−2.045	Catalase
PA4890 TR	*desT*	−1.595			DesT
PA4944	*hfq*			1.742	Hfq

a Gene number, gene name and product name are from the Pseudomonas Genome Project (http://www.pseudomonas.com). Genes previously reported to be QS-controlled are in bold (Hentzer *et al*., 2003; Schuster *et al*., 2003; Wagner *et al*., 2003).

* indicates genes regulated by MvfR (PqsR) in Déziel and colleagues (2005);

** indicate genes regulated by PQS in Bredenbruch and colleagues (2006);

TR known or predicted transcriptional regulator; RND, resistance-nodulation-cell division.

b Fold change in gene expression of *P. aeruginosa* PAO1 wild type (wt) compared with *P. aeruginosa* PAO1 *pqsA* mutant (*pqsA*).

c Fold change in gene expression of *P. aeruginosa* PAO1 wild type (wt) compared with *P. aeruginosa* PAO1 *pqsE*ind strain (*pqsE*ind).

d Fold change in gene expression of *P. aeruginosa* PAO1 wild type (wt) compared with *P. aeruginosa* PAO1 *pqsE*ind strain grown in presence of 1 mM IPTG (*pqsE*ind+IPTG).

Among the 54 genes upregulated in the *pqsA* mutant were those involved in the catabolism of catechol and anthranilate (*catA*, *catB*, *catC*), in anaerobic respiration (*narL*, *narG*), and in biofilm formation (*cupA1*) (Table 1; Vallet *et al*., 2001; Oglesby *et al*., 2008; Toyofuku *et al*., 2008), suggesting that the products of the *pqs* operon have a negative impact on the expression of certain genes. The upregulation of the *cat* genes in the *pqsA* mutant with respect to the wild-type strain is most likely due to an accumulation of anthranilate from the inability of the *pqsA* mutant to convert this compound into AQs (Bredenbruch *et al*., 2005). The upregulation of the *nar* genes observed in the *pqsA* mutant is consistent with the negative effect exerted by PQS on denitrification in *P. aeruginosa* (Toyofuku *et al*., 2008).

The observation that genes coding for known (*xylS*, *desT*, *soxR*, *pchR*) or predicted (PA0236, PA1196, PA1603) transcriptional regulators are both up- and down-regulated in the *pqsA* mutant (Table 1) suggests that the AQ stimulon is enlarged through the action of multiple auxiliary regulators.

### Characterization of the *pqsE* stimulon

The PAO1 *pqsA* mutant does not produce any AQs, therefore in this strain the PQS- and HHQ-dependent activation of the *pqsABCDE* operon is abrogated (Diggle *et al*., 2007). However, while the exogenous provision of either PQS or HHQ restored *pqsA* expression in the *pqsA* mutant (or in the *pqsAH* mutant which cannot convert exogenous HHQ to PQS; Diggle *et al*., 2007) only PQS fully induces the expression of PAO1 target genes such as *lecA* (Diggle *et al*., 2007). As *pqsE* is required for Lectin A production, these data imply that there may be differential regulation of *pqsE* by PQS and HHQ, i.e. exogenous PQS is capable of driving the expression of the entire *pqsABCDE* operon more efficiently than HHQ. To determine whether this was the case, RT-PCR was used to evaluate the levels of the *pqsE* transcript in *pqsA* and *pqsAH* mutants in the presence or absence of PQS or HHQ or both. Only a basal level of the *pqsE* gene transcript was detected in the *pqsA* mutant when grown in absence of HHQ or PQS (Appendix S1 and Fig. S1B). The addition of these AQs, either individually or in combination, restored the transcription of *pqsE*, both in the *pqsA* mutant and in the *pqsAH* double mutant, confirming that either HHQ or PQS or both can trigger the transcription of the entire *pqs* operon including *pqsE* (Appendix S1 and Fig. S1B; Diggle *et al*., 2007).

**Fig. 1:**
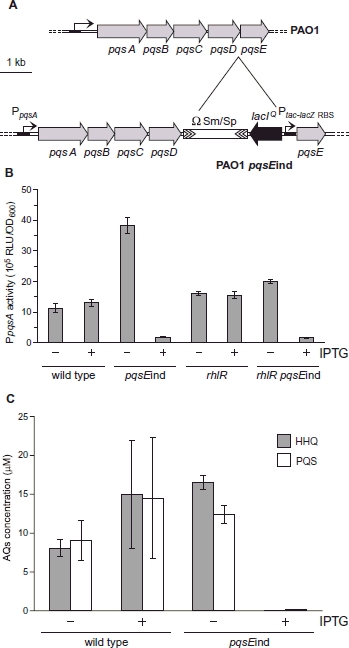
A. Schematic representation of the *pqs* locus in *P. aeruginosa* PAO1 wild type and the IPTG-inducible *pqsE* strain, *pqsE*ind. The Ω interposon (Sm^R^/Spc^R^) is from plasmid pHP45Ω: the *lacI^Q^* repressor is derived, together with the P*tac* promoter, from plasmid pME6032. B. Activity of the P*pqsA*::*lux* promoter fusion. The activity of the P*pqsA* promoter was monitored during growth in PAO1 wild type, *pqsE*ind, *rhlR* and *pqsE*ind *rhlR* double mutants. The maximal expression levels reached during the late exponential phase of growth are shown. Where indicated (+), 1 mM IPTG was added to the growth medium. Error bars are calculated from three independent experiments. C. Concentration of HHQ (grey bars) and PQS (white bars) determined by LC-mass spectrometry in PAO1 wild type and the *pqsE*ind mutant. The AQs were extracted from overnight cultures grown in LB broth; where indicated (+), 1 mM IPTG was added to the growth medium. The average of the results from three independent experiments is shown and error bars represent two standard deviations from the mean.

As *pqsE* influences the production of secondary metabolites such as pyocyanin and the rhamnolipids (Gallagher *et al*., 2002; Diggle *et al*., 2007; Farrow *et al*., 2008), it was not possible to determine whether the differentially regulated genes in the *pqsA* stimulon were affected as a consequence of a lack of AQs or PqsE. To address this question, we constructed a *P. aeruginosa* strain (*pqsE*ind) in which the chromosomal *pqsE* gene was placed under the control of an IPTG-inducible promoter, such that the expression of *pqsE* is independent from the *pqsABCD* genes (Fig. 1A). The levels of *pqsE* expression in *pqsE*ind and wild-type strains were compared by qRT-PCR analysis at an OD_600_ of 0.5 (early exponential phase) and 1.5 (late exponential phase). In both cases, when grown in the absence of IPTG the *pqsE*ind strain expressed *pqsE* only at a basal level, while the provision of IPTG resulted in the premature induction and overexpression of *pqsE* with respect to the parental strain. *pqsE* transcription increased 9.6-fold at an OD_600_ 0.5, and 18.1-fold at an OD_600_ 1.5 with respect to the wild-type strain at the same OD_600_ (Appendix S1 and Fig. S2). The growth curve of each of the strains evaluated was identical with or without IPTG (data not shown).

The transcriptional profiles of the PAO1 wild type and isogenic PAO1 *pqsE*ind strains were compared in the presence and absence of 1 mM IPTG during the late exponential phase of growth (OD_600_ 1.5). The control microarray analysis in which the wild-type PAO1 strain was grown with or without IPTG demonstrated that IPTG by itself had a negligible effect on the *P. aeruginosa* transcriptome (data not shown). A comparison of the parent strain and PAO1 *pqsE*ind grown without IPTG revealed that *pqsE* mutation altered the expression of 57 genes (Appendix S1 and Table S1); 51% of these genes were also altered in the *pqsA* mutant transcriptome, indicating that the *pqsE* stimulon comprises a subset of the *pqsA* stimulon. Consequently many of the changes in gene expression noted in the *pqsA* transcriptome are likely to be due to a lack of *pqsE* expression in the *pqsA* mutant. However, 49% of the genes affected in the *pqsE*ind strain were not present in the *pqsA* stimulon (Appendix S1 and Table S1). This finding suggests that these genes are differentially regulated by the AQs and PqsE, such that the loss of *pqsE* expression alone in the *pqsE* mutant disrupts the regulatory homeostasis so that they are apparent in the *pqsE* but not in the *pqsA* microarray, where the AQs and PqsE are absent.

PQS is a potent iron chelator and the addition of exogenous PQS to the wild type or to a *pqsA* mutant strongly induces genes involved in pyochelin and pyoverdin iron transport (Bredenbruch *et al*., 2006; Diggle *et al*., 2007). Conversely, compared with the wild type, mutation of either *pqsA* or *pqsE* results in the downregulation of pyochelin siderophore biosynthesis, regulatory and transport genes (*pchA*, *pchB*, *pchD*, *pchE*, *pchF*, *pchI*, *pchR* and *ftpA*; Table 1). These data suggest that the loss of PQS and its iron-chelating properties is not the sole determinant for the downregulation of the pyochelin biosynthetic pathway, and that PqsE may either play a role in iron homeostasis or in an as yet unidentified function of pyochelin unrelated to iron transport.

When compared with the wild-type parent strain, the transcriptional profile of the *pqsE*ind strain grown in presence of 1 mM IPTG (which results in premature induction and overexpression of *pqsE*) revealed the differential expression of 237 genes; 133 genes were upregulated and 104 genes were downregulated. Apart from genes involved in pyocyanin and pyochelin production, in this experimental setting, PqsE also affected the expression of genes involved in type II secretion (*xcpS*, *xcpV*, *xcpW*, *xcpY*, *xcpZ*), flagellar biosynthesis (*fliE*, *fliI*, *flhA*) and the post-transcriptional regulator *hfq* (Table 1; Appendix S1 and Table S1). Of these, the most downregulated genes are those involved in pyochelin biosynthesis (*pchA*, *pchD*, *pchE* and *pchF*), transport (*pchI*, *fptA*) and regulation (*pchR*). Compared with the wild type, these genes were also downregulated in the *pqsA* mutant and in the *pqsE*ind strain in the absence of IPTG. Thus either a lack of PqsE and PQS or the premature induction/overexpression of *pqsE* (where no PQS is produced) results in the downregulation of pyochelin genes.

The *pqsABCDE* operon was also present among the genes repressed as a consequence of *pqsE* overexpression (Table 1). This was of particular interest considering that it has previously been reported that mutation of *pqsE* did not affect the production of PQS (Gallagher *et al*., 2002). Surprisingly, the *pqsE* gene itself was not present among the *pqsE*-regulated genes. Considering that the qRT-PCR analysis performed on the same strains grown under the same experimental conditions clearly show marked changes in *pqsE* expression (Appendix S1 and Fig. S2), these data highlight the limitations intrinsic to high-density oligonucleotide microarrays and suggest that some genes may escape our analysis. Nevertheless, these data demonstrate that PqsE is a key player in the QS-dependent adaptive behaviour of *P. aeruginosa*.

### PqsE can regulate *pqsA* expression and AQ production

To investigate further the impact of *pqsE* on the transcription of the *pqsABCDE* genes, we measured the activity of the *pqsA* promoter (P*pqsA*) via fusion with *lux* reporter genes in the *P. aeruginosa* PAO1 wild type and *pqsE*ind strains, grown in presence and absence of IPTG. As shown in Fig. 1B, the activity of P*pqsA* is increased in the absence of *pqsE* and severely reduced when *pqsE* is overexpressed. The strong repression of P*pqsA* activity resulted in the inability of the *pqsE* overexpressing strain to produce HHQ and PQS (Fig. 1C) and other AQs (data not shown), while the concentrations of the AHLs, *N*-(3-oxododecanoyl)homoserine lactone (3-oxo-C12-HSL) and C4-HSL were unaffected (data not shown). As *pqsE* expression is dependent on PQS and HHQ, the ability of PqsE to reduce AQ biosynthesis through downregulation of *pqsABCDE* operon reveals that PqsE generates an autoregulatory negative-feedback loop.

Farrow and colleagues (2008) reported that PqsE regulates the production of pyocyanin and rhamnolipids in a RhlR/C4-HSL-dependent manner (Farrow *et al*., 2008). To determine whether the *pqsE*-dependent regulation of P*pqsA* is also mediated via RhlR, we determined the activity of P*pqsA* in a *P. aeruginosa* PAO1 *rhlR pqsE*ind double mutant. Figure 1B shows that *pqsE* induction can still abrogate P*pqsA* activity in the absence of *rhlR*, suggesting that the mechanism of action of PqsE in this context is RhlR-independent.

### PqsE is required for swarming motility and biofilm formation

As PqsE can function in an AQ-independent manner as well as modulating AQ biosynthesis, we examined the contribution of PqsE to pyocyanin and lectin production, motility and biofilm development in the *pqsE*ind mutant and also in a *pqsA pqsE*ind double mutant with and without induction since the latter permitted us to evaluate the impact of PqsE in the absence of AQs.

**Fig. 2:**
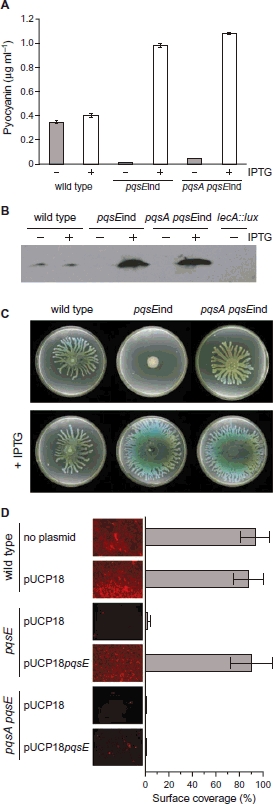
A. Pyocyanin produced by PAO1 and both *pqsE*ind and *pqsA pqsE*ind mutants. Bacterial cultures were grown in LB broth (grey bars) or LB broth supplemented with 1 mM IPTG (white bars), and pyocyanin was extracted after 16 h growth (early stationary phase). B. Western blot analysis of Lectin A in cell extracts of PAO1 and both *pqsE*ind and *pqsA pqsE*ind mutants. Proteins were extracted from cultures grown for 16 h in LB broth to an OD_600_ of 2.5 (early stationary phase of growth), with (+) or without (−) 1 mM IPTG. An extract from *P. aeruginosa* PAO1 *lecA* mutant (*lecA*::*lux*) was used as a negative control. C. Swarming assays performed with PAO1 and both *pqsE*ind and *pqsA pqsE*ind mutants in the presence or absence of 1 fmM IPTG. D. Biofilm formation on stainless steel coupons by PAO1 and both *pqsE* and *pqsA pqsE*ind mutants. A representative picture of the biofilm formed by each strain is also shown. For A and D, the average of the results from three independent experiments is reported with standard deviations.

Figure 2A and B shows that neither PAO1 *pqsEind* nor PAO1 *pqsA pqsE*ind produce much pyocyanin or lectin when *pqsE* is not induced, while IPTG-dependent *pqsE* overexpression results in the production of almost 2.5 times the wild-type level of pyocyanin and substantially higher levels of Lectin A in both mutants, demonstrating that PqsE controls both virulence determinants in an AQ-independent manner. Despite the differential production of pyocyanin and Lectin A, the microarray data (Table 1) did not reveal major changes in the *phz* or *lecA* transcript levels in the *pqsE*-inducible strain compared with the wild type. This could either reflect a possible post-transcriptional regulatory role for PqsE or highlight an experimental limitation of the microarray technique employed.

As our microarray data indicate that premature induction/overexpression of *pqsE* also modulates genes involved in motility such as *tadC*, *fliE*, *fliI*, *flhA* (Table 1; Tomich *et al*., 2007; Barken *et al*., 2008), we investigated the effect of *pqsE* on swimming, swarming and twitching motility. Twitching mainly relies on type IV pili (Mattick, 2002), while swimming motility is flagellar-mediated. Although some genes involved in flagellar motility were regulated by *pqsE* in the microarray experiments (*fliE*, *fliI*, *flhA*), agar plate motility assays indicated that *pqsE* did not affect swimming or twitching under the experimental conditions employed (data not shown). Conversely, the swarming plate assays demonstrated that *pqsE* is required for swarming, because the *pqsE*ind mutant failed to swarm in the absence of IPTG (Fig. 2C). However, swarming was restored to wild-type levels in the *pqsA pqsE*ind double mutant in the absence of IPTG. In both cases, the IPTG-induced expression of *pqsE* resulted in a ‘hyper-swarming phenotype’ (Fig. 2C). The inability of the *pqsE*ind single mutant to swarm in the absence of IPTG implies that the AQs may inhibit swarming in the absence of PqsE.

As motility is often inversely related to biofilm formation (Verstraeten *et al*., 2008), we examined the effect of *pqsE* mutation on the ability of *P. aeruginosa* to form biofilms on stainless steel. As IPTG disrupts biofilm formation in *P. aeruginosa* (Diggle *et al*., 2006), we performed these experiments using isogenic PAO1 *pqsE* and PAO1 *pqsA pqsE* deletion mutants, carrying either the pUCP18 vector plasmid, or the pUCP*pqsE* plasmid for *pqsE* complementation. As shown in Fig. 2D, PqsE is required for biofilm formation, but in this case the expression of *pqsE* in the *pqsA pqsE*ind mutant failed to restore biofilm production to wild-type levels. Exogenously added PQS partially restored biofilm formation (data not shown), indicating that both *pqsE* and PQS are required for the development of a mature biofilm.

### PqsE restores virulence in the absence of AQs in nematode, plant and murine infection models

**Fig. 3:**
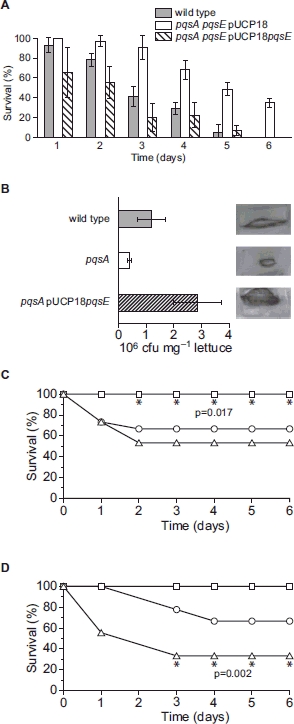
PqsE restores virulence in nematode, plant and animal infection models in the absence of AQs. A. *Caenorhabditis elegans* killing assay showing the percentage of nematode survival after 1–6 days of exposure to the *P. aeruginosa* PAO1 wild type, *pqsA pqsE*ind mutant and *pqsA pqsE*ind mutant transformed with the vector control, pUCP18 or pUCP*pqsE* respectively. The average of four independent experiments is reported with standard deviation. B. Virulence of the wild type, *pqsA* mutant and *pqsA* mutant complemented with *pqsE* in the lettuce leaf virulence assay. The number of bacterial cells (as colony forming units, cfu) present in 1 mg of lettuce midrib 5 days post injection is shown. Error bars were calculated from five independent experiments. A representative picture of infected midribs is also shown for each strain. C and D. Mouse acute burn wound infection showing the survival rate over time (days after burn/infection) for mice infected with (C) the *P. aeruginosa* wild type (▵), *pqsA* (□) and *pqsE* (○) mutants; 15 mice per mutant and (D) the *P. aeruginosa pqsA* mutant (□) and the *pqsA* mutant transformed with either pUCP18 (○) or pUCP*pqsE* (▵); nine mice per mutant.

Given that PqsE restores pyocyanin and lectin production in the absence of AQs, we investigated the impact of *pqsE* mutation on *P. aeruginosa* pathogenicity using three well-established plant (lettuce leaf) and animal (*Caenorhabditis elegans* and mouse) experimental infection models. In these experiments, we compared the virulence of the PAO1 wild type, *pqsE* mutant and *pqsA pqsE* double mutant transformed with either the pUCP18 or pUCP*pqsE* vectors. The data obtained from each infection model are shown in Fig. 3. In the *C. elegans* model (Fig. 3A), PAO1 killed all of the nematodes after 6 days compared with ∼65% of worms fed with the PAO1 *pqsA pqsE* mutant. Expression of plasmid-borne *pqsE* in the *pqsA pqsE* mutant fully restored virulence. Similar results were also obtained for the *pqsE* mutant that was less virulent than the wild type unless complemented with *pqsE* (data not shown). Furthermore, *C. elegans* fed with the PAO1 *pqsA pqsE* pUCP*pqsE* strain exhibited symptoms of sickness, including impaired locomotion much faster (within 1 day) than was observed for *C. elegans* fed with the wild type (3 days). These worms also showed a significant reduction in fertility.

When inoculated into the mid-ribs of lettuce leaves, the PAO1 *pqsA* mutant causes much less tissue necrosis and grows more poorly than the wild-type strain (Fig. 3B). Transformation of this mutant with plasmid-borne *pqsE* fully restored virulence.

In a mouse burn wound infection model, the *pqsA* mutant is highly attenuated compared with the *pqsE* mutant that in turn is less virulent than the wild type (Fig. 3C). Introduction of the *pqsE*-expressing plasmid into the *pqsA* mutant strain completely restored virulence also in this infection model (Fig. 3D).

## Discussion

**Fig. 4:**
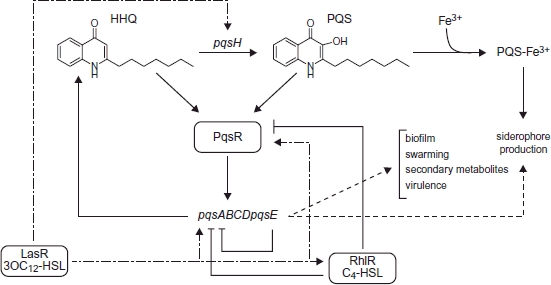
Simplified schematic representation of the AQ-dependent QS in *P. aeruginosa* (modified from Diggle *et al*., 2007). HHQ, the immediate precursor of PQS, drives the expression of the *pqsABCDE* operon via PqsR(MvfR) and is also converted to PQS by the action of the monooxygenase, PqsH. PQS also drives *pqsABCDE* expression via PqsR. PqsE positively regulates biofilm, swarming virulence and secondary metabolite gene expression but negatively regulates *pqsABCDE* expression. PQS also binds ferric iron which results in the induction of high affinity siderophore iron transport genes. AHL and AQ-dependent QS are linked because LasR/3-oxo-C12-HSL is required for maximal expression of *pqsH* and *pqsR* whereas *pqsR* and *pqsABCDE* are repressed by RhlR/C4-HSL.

*Pseudomonas aeruginosa* employs a sophisticated multi-signal molecule QS system which operates to facilitate environmental adaptation at the population level (Fig. 4). To further refine our understanding of the individual contributions of key components of the AQ signalling pathway to *P. aeruginosa* physiology, we first determined the extent of the *P. aeruginosa* PAO1 *pqsA* stimulon, because this has not previously been reported. By profiling the transcripts present after maximal induction of the *pqsABCDE* operon, we observed that 158 genes were up- or down-regulated when the wild type was compared with the *pqsA* mutant strain (Table S1). However, only 18 and 21 of these genes were previously identified in transcriptome analyses of either (i) a *P. aeruginosa* PA14 *pqsR* (*mvfR*) mutant (Déziel *et al*., 2005) or (ii) *P. aeruginosa* PAO1 grown in the presence of exogenous PQS added at the point of inoculation (Bredenbruch *et al*., 2006) and compared with the corresponding wild-type strains. These variations are perhaps not particularly surprising given the differences in the strains and experimental conditions used. Although the *pqsR* mutant in common with the *pqsA* mutant is AQ-negative, it is not known whether PqsR directly drives the expression of target genes other than *pqsA*. Furthermore, the exogenous addition of PQS to the wild-type *P. aeruginosa* (which is already producing AQs in a population density dependent manner) is likely to advance and enhance PQS-dependent gene expression (Diggle *et al*., 2003), as well as inducing an oxidative/anti-oxidative stress response (Bredenbruch *et al*., 2006; Häussler and Becker, 2008). However, despite these differences, the recurrence of the same set of genes in at least two out of the three experiments, indicates that AQ-dependent QS plays a key role in regulating genes of known function including *pqsABCDE*, *phnAB*, *phzABCDEFG*, *mexGHIompD*, *pchABCDEF*, *lecA*, *chiC* and *pvdH*, as well as many others of unknown function.

In contrast to *pqsA* mutants, *pqsE* mutants produce AQs such as PQS and HHQ but make very little pyocyanin, elastase, Lectin A or rhamnolipid (Gallagher *et al*., 2002; Diggle *et al*., 2003). This raises the question as to whether the differentially regulated genes in the *pqsA* mutant were affected as a consequence of a lack of AQs or PqsE. By constructing an inducible *pqsE* strain (*pqsE*ind), we determined the extent of the PqsE stimulon by comparing the transcriptional profiles of the PAO1 wild-type strain with the uninduced or induced *pqsE*ind strain, in which *pqsE* was not expressed or prematurely induced and overexpressed respectively. The results obtained revealed that PqsE regulates a major subset of the AQ-controlled genes, including those involved in siderophore biogenesis even in the absence of the iron-chelating properties of PQS. The observation that pyochelin genes are upregulated in the wild-type strain with respect to both the induced and the uninduced *pqsE*ind strains suggests that a fine balancing of PQS and PqsE is required to produce physiologically profitable levels of this siderophore. It is likely that both PQS and PqsE have a positive effect on pyochelin production, as suggested by the reduced expression of genes involved in the synthesis of this siderophore in the *pqsA* and *pqsE*ind strain with respect to the wild-type strain (Table 1). However, as the pyochelin genes are also downregulated when *pqsE* is overexpressed (*pqsE*ind with IPTG; Table 1), this can be explained by the complete loss of PQS production in this strain (Fig. 1C).

The greater number of differentially expressed genes observed when *pqsE* is prematurely induced and overexpressed may be partially explained by the high level of pyocyanin produced under these conditions. This is because pyocyanin, in addition to functioning as a redox reactive virulence determinant (Lau *et al*., 2004), also acts as a terminal signal molecule for a subset of QS-dependent genes, including the *mexGHI-ompD* efflux pump and PA2274 (Dietrich *et al*., 2006). Pyocyanin is also capable of inducing oxidative stress, which is consistent with the upregulation of genes such as *ahpF* (alkylhydroperoxidase), *katA* and *katE* (catalase genes) when *pqsE* is induced. Interestingly, SoxR, which is required for the pyocyanin-dependent upregulation of the *mexGHI-ompD* pump, is downregulated in both the *pqsA* and *pqsE* mutant arrays, as well as the six genes which make up the SoxR regulon (PA2274, *mexGHI-ompD* and PA3718; Palma *et al*., 2005) (Table 1 and Table S1). Although SoxR is not a key regulatory player in the oxidative stress response of *P. aeruginosa*, it is essential for virulence in a mouse lung infection model (Palma *et al*., 2005). Given that PqsE regulates *soxR* and hence its regulon, this suggests that the contribution of the SoxR regulon to virulence is controlled by the AQs and PqsE.

We have shown that PqsE can repress the transcription of the *pqsABCDE* operon (and consequently its own transcription). This autoregulatory role of PqsE is analogous to the homeostatic effect exerted by RsaL on the production of 3-oxo-C12-HSL in *P. aeruginosa*. RsaL transcription is dependent on the LasR/3-oxo-C12-HSL complex. When exceeding a certain physiological concentration, RsaL binds to the *rsaL*-*lasI* bidirectional promoter repressing the transcription of both genes. This regulatory circuit generates an incoherent feed-forward loop that provides 3-oxo-C12-HSL homeostasis (Rampioni *et al*., 2006; 2007;). Similarly, PqsE requires the PqsR/HHQ or PqsR/PQS complex for its transcription, but when induced independently and prematurely, PqsE represses the expression of *pqsABCDE*, and hence AQ synthesis and its own transcription. Furthermore, in an *rsaL* mutant, the levels of 3-oxo-C12-HSL increase steadily whereas no AQ accumulation was observed in the *pqsE* mutant over the growth curve (data not shown). This difference may be ascribed to a limiting concentration of the substrates required for AQ biosynthesis, or due to the involvement of other, as yet unidentified factors regulating the transcription of the *pqsABCDE* operon.

While RsaL is a transcriptional regulator, PqsE is an enzyme which lacks any obvious DNA-binding domains, indicating that this protein is likely to exert its regulatory role indirectly. The structure of PqsE has been determined and the presence of the predicted metallo-β-lactamase fold with a metal centre in the active site confirmed (Yu *et al*., 2009). While neither the true substrate for PqsE nor the downstream signal transduction pathway has yet been identified, the purified recombinant PqsE protein is capable of slowly hydrolysing phosphodiesters, nucleic acids and thioesters (Yu *et al*., 2009).

Determination of the *pqsE* stimulon, the physiological characterization of the *pqsE* mutant and the consequences of expressing *pqsE* in a *pqsA* mutant demonstrate the central importance of PqsE within the AQ-signal transduction pathway and its capacity for acting independently of PQS and HHQ. While the action of PqsE in restoring pyocyanin production and *rhlA* expression is dependent on RhlR/C4-HSL (Farrow *et al*., 2008), this is not the case for the PqsE-mediated repression of *pqsA*, which is independent of the *rhl* system (Fig. 1B). Consequently PqsE may activate or repress target genes via distinct pathways.

The *P. aeruginosa pqsE*ind mutant failed to swarm in the absence of IPTG and exhibited a hyper-swarming phenotype following induction. This result is in line with the previous finding that *pqsE* is involved in rhamnolipid production (Diggle *et al*., 2003). Surprisingly, swarming motility in the *pqsA pqsEind* double mutant was restored (Fig. 2C), implying a role for AQs in repressing swarming in the absence of PqsE. This phenotype may be explained by the function of PQS as an activator of the small regulatory RNA, RsmZ (S. Heeb, K.M. Righetti, M. Messina, S.A. Kuehne, C. Pustelny, S.R. Chhabra *et al*., unpublished data), which titrates out the RNA-binding protein RsmA (S. Heeb, K.M. Righetti, M. Messina, S.A. Kuehne, C. Pustelny, S.R. Chhabra *et al*., unpublished data). A PAO1 *rsmA* mutant is unable to swarm (Heurlier *et al*., 2004), and therefore mutation of *pqsA* (which results in the loss of PQS) would result in a reduction in RsmZ, consequently increasing the availability of RsmA and so restoring swarming motility. It is also possible that PqsE is involved in the post-transcriptional regulation of pyocyanin and Lectin A because only minor changes in *phz* or *lecA* transcript levels were noted in the *pqsE*ind mutant in the presence or absence of IPTG despite major changes in pyocyanin and Lectin A protein levels. The presence of the post-transcriptional regulator *hfq* among the genes regulated by PqsE (Table 1) supports this possibility.

As well as an inability to swarm, the *pqsE* mutant was unable to form mature biofilms on a stainless steel substratum. However, in contrast to pyocyanin and lectin, *pqsE* was not sufficient to restore biofilm formation in a *pqsA* mutant background (Fig. 2). This is likely to be because the release of extracellular DNA which is crucial for biofilm maturation is *pqsA*-dependent (Allesen-Holm *et al*., 2006; Yang *et al*., 2007), and also because PQS induces oxidative DNA damage leading to DNA release and fragmentation in growing *P. aeruginosa* cultures (Häussler and Becker, 2008). It is therefore likely that the development of mature biofilm requires both the expression of PqsE and the production of PQS. Additionally, biofilm development is a complex pleiotropic phenotype and the impact of QS on this process is strongly dependent upon experimental conditions and growth media (Kirisits and Parsek, 2006). However, the induction of genes involved in iron sequestration observed in the *pqsE* mutant is indicative of iron starvation conditions, which have been reported to influence biofilm formation negatively (Banin *et al*., 2005), consistent with the reduced biofilm forming capacity of the *pqsE* mutant.

*Pseudomonas aeruginosa* PA14 strains carrying mutations in *pqsR*, *pqsA*, *pqsB* and *pqsE* have previously been reported to display reduced virulence in a mouse burn wound model (Déziel *et al*., 2005). Here we have confirmed these data for the PAO1 *pqsA* mutant which was completely avirulent. However, transformation of the *pqsA* mutant with the plasmid-borne *pqsE* gene fully restored virulence indicating that the AQs are dispensible for virulence. Similar results were obtained in both *C. elegans* and lettuce leaf infection models. Because these are acute infection models and given that PQS is essential for biofilm maturation, it is tempting to speculate that PqsE is essential for acute infections, while the AQs may play a more important role in chronic biofilm-centred infections. In this context, the autorepressive regulatory circuit generated by PqsE may play an important role in fine tuning the levels of AQ- and PqsE-regulated virulence factors.

Although the precise function of PqsE remains enigmatic, the work described here clearly demonstrates that PqsE is a global regulator within the QS network (Fig. 4) which plays a pivotal role in controlling the adaptive behaviour of *P. aeruginosa*.

## Experimental procedures

### Bacteria, growth conditions and plasmids

The bacterial strains and plasmids used are listed in Table 2. *Escherichia coli* and *P. aeruginosa* strains were routinely grown in Luria–Bertani (LB) broth. All strains were grown at 37°C in 10 ml of broth and 100 ml flasks with shaking at 200 r.p.m. The following reagents were added as required: ampicillin (Ap) 100 µg ml^−1^; nalidixic acid 15 µg ml^−1^; chloramphenicol (Cm) 30 µg ml^−1^ (*E. coli*) or 375 µg ml^−1^ (*P. aeruginosa*); tetracycline (Tc) 10 µg ml^−1^ (*E. coli*) or 200 µg ml^−1^ (*P. aeruginosa*); carbenicillin (Cb) 400 µg ml^−1^; streptomycin (Sm) 800 µg ml^−1^; IPTG 1 mM; PQS or HHQ (40 µM; synthesized as described before by Diggle *et al*., 2006). To select *P. aeruginosa* from *E. coli* after mating experiments, LB agar plates supplemented with nalidixic acid 15 µg ml^−1^ were used.

**Table 2:** Bacterial strains and plasmids used in this work.

Strain/plasmid	Relevant characteristics	Source/reference
Strain		
*E. coli*		
DH5α	Cloning strain	Grant *et al*. (1990)
S17.1λ*pir*	Conjugative strain for suicide plasmids.	Simon *et al*. (1983)
*P. aeruginosa*		
PAO1	Nottingham collection wild-type *P. aeruginosa* strain	
*pqsA*	*pqsA* mutant of PAO1	Aendekerk *et al*. (2005)
*pqsA pqsH*	*pqsA pqsH* double mutant of PAO1	Diggle *et al*. (2007)
*lecA*::*lux*	*lecA* mutant of PAO1	Winzer *et al*. (2000)
*pqsE*ind	PAO1 derivative in which *pqsE* expression is under the control of a P*tac* promoter	This study
*pqsE*	*pqsE* in-frame deletion mutant of PAO1	This study
*rhlR*	*rhlR* in-frame deletion mutant of PAO1	This study
*pqsA pqsE*ind	PAO1 *pqsA* mutant derivative in which *pqsE* expression is under the control of a P*tac* promoter	This study
*rhlR pqsE*ind	PAO1 *rhlR* mutant derivative in which *pqsE* expression is under the control of a P*tac* promoter	This study
*pqsA pqsE*	*pqsA pqsE* double mutant of PAO1	This study
Plasmid		
pBluescript-II KS+	Cloning vector; ColE1 replicon; Ap^R^	Stratagene
pUCP18	pUC18-derivative containing a stabilising fragment for maintenance in *Pseudomonas*; Ap^R^, *E. coli*/Cb^R^, *P. aeruginosa*.	Schweizer (1991)
mini-CTX*lux*	Promoter-probe vector containing the *luxCDABE* operon; Tc^R^	Becher and Schweizer (2000)
pDM4	Suicide vector; *sacBR*, *oriR6K*; Cm^R^	Milton *et al*. (1996)
mini-CTX*pqsA*::*lux*	Plasmid to insert P*pqsA*-*lux* fusion into the chromosome of *Pseudomonas*; Tc^R^	Diggle *et al*. (2007)
pDM4Δ*pqsE*	pDM4 derivative for *pqsE* in-frame deletion; Cm^R^	This study
pDM4Δ*rhlR*	pDM4 derivative for *rhlR* in-frame deletion; Cm^R^	This study
pDM4*pqsE*ind^a^	pDM4 derivative for the generation of the *pqsE*-inducible strain; Cm^R^	This study
pUCP*pqsE*	pUCP18 derivative for *pqsE* complementation; Ap^R^	This study
pBS*rhlR*Up	pBluescript-II KS+ derivative containing the upstream region of *rhlR*; Ap^R^	This study
pBS*rhlR*Dw	pBluescript-II KS+ derivative containing the downstream region of *rhlR*; Ap^R^	This study
pBS*pqsE*Up	pBluescript-II KS+ derivative containing the upstream region of *pqsE*; Ap^R^	This study
pBS*pqsE*Dw	pBluescript-II KS+ derivative containing the downstream region of *pqsE*; Ap^R^	This study

a More details on the construction of *P. aeruginosa pqsE*ind strain are given in *Supporting Information*.

### DNA manipulation

Oligonucleotides used in this study are listed in Table S2 and Appendix S1. Plasmid DNA preparations, restriction enzyme digestions, agarose gel electrophoresis and ligations were performed using standard methods (Sambrook and Russell, 2001). Transformation of *P. aeruginosa* was carried out by electroporation as published (Farinha and Kropinski, 1990). Routine DNA sequencing was conducted in the DNA Sequencing Laboratory, Biopolymer Synthesis and Analysis Unit, Queens Medical Centre, University of Nottingham.

### Construction of *P. aeruginosa* PAO1 mutants

The PAO1 *rhlR* and *pqsE* chromosomal deletion mutants were constructed by allelic exchange using the suicide vector pDM4Δ*rhlR* and pDM4Δ*pqsE* respectively. pDM4Δ*rhlR* and pDM4Δ*pqsE* were made as follows; using PAO1 template DNA, upstream fragments of the *rhlR* and *pqsE* genes were amplified using the primers *rhlR*Up1 and *rhlR*Up2 (for *rhlR*) or *pqsE*Up1 and *pqsE*Up2 (for *pqsE*), while downstream fragments were amplified using the primers *rhlR*Dw1 and *rhlR*Dw2 (for *rhlR*) or *pqsE*Dw1 and *pqsE*Dw2 (for *pqsE*). The resulting PCR products were cloned in pBluescript-II KS+, resulting in the plasmids pBS*rhlR*Up and pBS*rhlR*Dw (for *rhlR*), and pBS*pqsE*Up and pBS*pqsE*Dw (for *pqsE*). The upstream and downstream fragments of each gene were then excised with the corresponding restriction enzymes and cloned in the suicide vector pDM4 (Milton *et al*., 1996), resulting in the plasmids pDM4Δ*rhlR* and pDM4Δ*pqsE*. Allelic exchange in *P. aeruginosa* PAO1 following conjugal mating with *E. coli* S17-1λ*pir* donor strains and sucrose counter-selection was performed as described by Westfall and colleagues (2004). The resulting PAO1 *rhlR* and PAO1 *pqsE* mutant strains were confirmed by PCR, sequence analysis and phenotypic assays.

The PAO1 *pqsE*ind strain was constructed by introducing onto the chromosome the *lacIQ* repressor gene and the *tac* promoter transcribing the *lacZ* 5′ untranslated transcribed region and its ribosome binding site (RBS) directly upstream of the *pqsE* open reading frame, resulting in strong, constitutive transcription and translation only in the presence of IPTG. An ΩSmR/SpR interposon (to terminate native transcription originating upstream of *pqsE*) and the *lacIQ* repressor gene were inserted upstream of the P*tac-lacZ* RBS-*pqsE* region (Fig. 1A). Further details are provided in Appendix S1.

To generate PAO1 *rhlR pqsE*ind and PAO1 *pqsA pqsE*ind double mutant strains, the pDM4*pqsE*ind suicide vector was conjugated in the PAO1 *rhlR* and PAO1*pqsA* mutants respectively, while to generate the PAO1*pqsA pqsE* double mutant strain, the pDM4Δ*pqsE* plasmid was conjugated in the PAO1*pqsA* mutant. In all cases and sucrose-resistant clones were selected and verified by PCR.

### Construction of pUCP*pqsE*

The *pqsE* gene was amplified from *P. aeruginosa* PAO1 chromosomal DNA using the primers *pqsE*18Fw and *pqsE*18RV. This PCR product was digested with SacI/XbaI and cloned into similarly digested pUCP18, resulting in the plasmid pUCP*pqsE*, which was verified by sequence analysis. The pUCP18 and pUCP*pqsE* plasmids were introduced into different *P. aeruginosa* PAO1 strains by electroporation (Farinha and Kropinski, 1990).

### RNA extraction and expression profiling experiments

*Pseudomonas aeruginosa* strains were grown at 37°C in 10 ml of LB broth and 100 ml Schott Duran flasks with shaking at 200 r.p.m. Where required, LB broth was supplemented with 1 mM IPTG. RNA was extracted from each culture at an OD_600_ of 1.5 (late exponential phase of growth). Cells were treated with RNAprotect Bacteria Reagent (Qiagen), and total RNA extraction was performed with the RNeasy Midi Kit (Qiagen) as per the manufacturer's instructions.

For the expression profiling experiments, the microarrays were designed to contain multiple oligonucleotide probes for all the PAO1 genes including the small RNA genes and were purchased from Oxford Gene Technology (Oxford, UK). For each array, 10 µg of RNA was reverse transcribed and directly labelled with Cy5-dCTP and 2 µg of genomic DNA was directly labelled with Cy3-dCTP. Samples were hybridized onto the arrays for 16 h. Scanning of the arrays was performed using the Axon 4000B GenePix Scanner, the data extraction software used was GenePix Pro 6, both from Molecular Devices (Sunnyvale, USA). For each strain, microarray experiments were performed in triplicate and data analysis performed using GeneSpring GX10 (Agilent Technologies, Santa Clara, USA). The array data underwent Lowess normalization, the most variable 10% of data according to standard deviation within replicates was removed and genes of altered expression were determined by passing through cut-offs of both a fold change of 1.5 and a paired *T*-test of *P* = 0.05.

### SDS-PAGE and immunoblotting

*Pseudomonas aeruginosa* cells grown in LB at 37°C overnight for 16 h to an OD_600_ 2.5 were lysed, normalized for protein content and subjected to SDS-PAGE prior to electrophoretic transfer to nitrocellose membranes and probed with a rabbit polyclonal antibody to Lectin A as described before (Diggle *et al*., 2003).

### Measurement of bioluminescence

The single copy fusion of the *pqsA* promoter to the *luxCDABE* genes was introduced in the different *P. aeruginosa* PAO1 strains by mating with the *E. coli* S17.1λ*pir* carrying the mini-CTX*pqsA*::*lux* plasmid (Diggle *et al*., 2007). Bioluminescence was determined as a function of population density by using an automated luminometer-spectrometer (TECAN). Overnight cultures of *P. aeruginosa* strains carrying the chromosomal *pqsA*::*lux* fusion were diluted 1:1000 in fresh LB broth, and 0.2 ml cultures were grown at 37°C in microtitre plates. Luminescence and turbidity were determined every 30 min. Luminescence is given as relative light units divided by OD_600_.

### Extraction and quantification of AQs

*Pseudomonas aeruginosa* strains were grown overnight in 10 ml of LB broth at 37°C. The AQs were extracted from 2 ml of the culture supernatant with 6 ml of acidified ethyl acetate (Diggle *et al*., 2003), vortexed vigorously and centrifuged at 9447 *g* for 5 min. The organic phase was transferred to a clean glass tube, dried to completion, resuspended in 50 µl methanol. Liquid chromatography (LC) was performed on an Agilent 1200 series HPLC with an Ascentis Express C18 150 × 2.1 mm internal diameter, 2.7 µm particle size, maintained at 50°C. The mobile phase consisted of formic acid 0.1% (v/v) in water and formic acid 0.1% (v/v) in acetonitrile run as a gradient over 20 min at a flow rate of 0.3 ml min^−1^. Using a Bruker HCT Plus ion trap LC-mass spectrometer in multiple reaction mode and Hystar software, ions were introduced, isolated and fragmented using positive ion electrospray. Retention times and MS2 peak spectra were matched to the 10 µM synthetic AQ standards injected at the beginning and end of each run.

### Pyocyanin production, motility and biofilm assays

For pyocyanin assays, *P. aeruginosa* strains were grown with aeration in LB at 37°C overnight for 16 h (to early stationary phase), and pyocyanin levels were determined in the supernatants derived from 5 ml of culture, as previously described (Xu *et al*., 2005). For motility assays, *P. aeruginosa* cultures (OD_600_ 1.0) were picked with a toothpick onto ‘Swimming Plates’ (1 g l^−1^ tryptone, 0.5 g l^−1^ yeast extract, 5 g l^−1^ NaCl, 3 g l^−1^ agar noble), or ‘Twitching Plates’ (10 g l^−1^ tryptone, 5 g l^−1^ yeast extract, 5 g l^−1^ NaCl, 10 g l^−1^ agar noble) respectively, and grown for 16 h at 37°C. For swarming assays, 2 µl of culture was spotted onto ‘Swarming Plates’ (5 g l^−1^ bacto agar, 8 g l^−1^ nutrient broth N°2, 0.5% (w/v) glucose) and grown 16 h at 37°C. Biofilm formation on stainless steel coupons was performed essentially as previously described (Diggle *et al*., 2006). Surface attached biofilms were stained with 0.1% (w/v) acridine orange, washed with PBS, air-dried and examined for bacterial attachment with an inverted fluorescent microscope (Nikon Eclipse TE200) using the ×40 objective lens and green filter. Ten images were collected per metal coupon using a JVC KY-F58 video camera. Sampling was conducted at random from the central portion of each coupon. Percentage surface coverage was calculated using the Lucia G/Comet software (Nikon UK). The assays were performed in triplicate for each strain.

### Virulence assays

*C. elegans* virulence assays were performed essentially as described by Papaioannou and colleagues (2009). *Caenorhabditis elegans* was incubated on bacterial lawns at 21°C on nematode growth medium (Stiernagle, 2006) and scored for live worms every day. For statistical purposes, four replicates with 10 worms for each strain were performed, and four replicates per trial were carried out with *E. coli* OP50 as negative control.

For the lettuce leaf virulence assay, 10 µl aliquots of bacterial cultures resuspended to an A_600_ of 0.1 in 10 mM MgSO_4_ were injected into the midribs of fresh Romaine lettuce leaves, and incubated for 2–5 days as previously described (Starkey and Rahme, 2009). Lettuce leaves were monitored for the appearance of soft-rot symptoms and the numbers of bacterial cells in the midrib were determined after a defined incubation period.

The mouse acute burn wound model described by Schaber and colleagues (2007) and by Rumbaugh and colleagues (2009) was carried out as follows. Female, Swiss Webster, mice were obtained from Charles River Laboratories (Wilmington, MA, USA). Mice used in experiments were 6–8 weeks old and weighed 20–25 g. Mice were anaesthetized, their backs were shaved and an acute scald wound induced as previously described, with infection doses of 10^2^ bacteria. Mice were housed and studied under protocols approved by the Institutional Animal Care and Use Committee in the animal facility of TTUHSC (Lubbock, TX).

## Supporting information

**Fig. S1.** RT-PCR analysis of the *pqsABCDE* operon.

A. Amplification of the individual *pqs* genes in PAO1. cDNA was synthesized from RNA extracted from *P. aeruginosa* PAO1 at various stages of growth (as determined by OD_600_). The corresponding RNA was amplified in parallel as a negative control, and the *P. aeruginosa* PAO1 genomic DNA was used as a positive control.

B. Amplification of the *pqsE* gene in PAO1 *pqsA* and PAO1 *pqsA pqsH* mutants. cDNA was synthesized from RNA extracted from cells grown to OD_600_ 1.5. Where indicated (+), strains were grown in presence of 40 μM HHQ and/or PQS. The corresponding RNA was amplified in parallel as a negative control, and the *P. aeruginosa* PAO1 genomic DNA was used as a positive control.

**Fig. S2.** Analysis of *pqsE* expression as a function of growth in the *pqsE*ind strain. The fold change in *pqsE* transcript levels in the pqsEind strain grown to early exponential phase (OD_600_ = 0.5) and late exponential phase (OD*600* = 1.5) was determined by qRT-PCR in the presence of absence of IPTG. The relative expression of *pqsE* is compared with that of the wild type at an OD_600_ of 0.5. Where indicated, IPTG (1 mM) was added to the growth medium.

**Table S1.** Genes regulated in the microarray experiments.

**Table S2.** Primers used in this work.

**Appendix S1.** Experimental procedures.
