# Supplementary material for: Transcriptomic analysis reveals a global alkyl-quinolone-independent regulatory role for PqsE in facilitating the environmental adaptation of Pseudomonas aeruginosa to plant and animal hosts
Source: Environ Microbiol. 2010 Jun;12(6):1659–73. doi: 10.1111/j.1462-2920.2010.02214.x (PMC2901523; doi:10.1111/j.1462-2920.2010.02214.x)
Supplement: Supplementary file 1 [file emi0012-1659-SD1.doc]

**Supporting Information**

**EXPERIMENTAL PROCEDURES**

**Construction of the *P. aeruginosa pqsE*ind strain**

The *P. aeruginosa* PAO1 *pqsE*ind was obtained by double crossover of the fragment carried by pDM4*pqsE*ind in *P. aeruginosa* PAO1 and selection of sucrose-resistant, Sm^R^ clones. The suicide plasmid pDM4*pqsE*ind was constructed in several stages and is a pDM4 derivative that carries, in sequential order, the following elements: (a) an *Xba*I-*Bam*HI fragment of 0.5 kb of the upstream region of *pqsE* obtained by PCR using primers EindFW1 and EindRV1; (b) the 2.0 kb *Bam*HI Sm/Spc integron from pHP45Ω; (c) the 1.5 kb *Bam*HI-*Eco*RI *lacI^Q^* P*tac* inducible promoter fragment of pME6032 and (d) an 0.5 kb *Eco*RI-*Xho*I fragment carrying the *pqsE* open reading frame obtained by PCR with primers EindFW2 and EindRV2 (the sequence of the primers is shown in Table S2, Supporting Information). Inserts obtained by PCR by amplification of PAO1 chromosomal DNA were verified by sequencing.

| **Strain/plasmid** | Relevant characteristics | Source/reference |
| --- | --- | --- |
| **Plasmid**  pME6032  pHP45Ω  pDM4*pqsE*ind | pVS1-p15A shuttle expression vector; Tc^R^  Source of ΩSm/Spc interposon  pDM4 derivative for the generation of the *pqsE* inducile strain; Cm^R^ | Heeb *et al*., 2002  Prentki & Krisch, 1984  This study |

Heeb, S., Blumer, C., and Haas, D. (2002) Regulatory RNA as mediator in GacA/RsmA-dependent global control of exoproduct formation in *Pseudomonas fluorescens* CHA0. *J Bacteriol* **184:**1046-1056.

Prentki, P., and Krisch, H.M. (1984) *In vitro* insertional mutagenesis with a selectable DNA fragment. *Gene* **29:**303-313.

**RT-PCR and qRT-PCR analyses**

*P. aeruginosa* strains were grown at 37°C in 10 ml of LB broth and 100 ml Schott Duran flasks with shaking at 200 r.p.m. Where required, LB broth was supplemented with 40 μM synthetic HHQ and/or PQS. RNA was extracted from each culture at the indicated OD_600_. Bacterial cells were treated with RNAprotect Bacteria Reagent (Qiagen), and total RNA extraction was performed with the RNeasy Mini Kit (Qiagen) by following the manufacturer’s instructions. RNA was digested with Turbo RNase-free DNAse (Ambion) and then retrotranscribed with Moloney Murine Leukemia Virus (M-MuLV) Reverse Transcriptase (New England BioLabs), according to manufacturer’s instructions.

To amplify the different target genes for the RT-PCR analysis, PCR was performed on the synthesized cDNA with the following primers: *pqsA*FW and *pqsA*RV for *pqsA*, *pqsB*FW and *pqsB*RV for *pqsB*, *pqsC*FW and *pqsC*RV for *pqsC*, *pqsD*FW and *pqsD*RV for *pqsD*, *pqsE*FW and *pqsE*RV for *pqsE* (the sequence of the primers is shown in Table S2, Supporting Information).

For the qRT-PCR analysis *pqsE* was amplified with primers *pqsE* q FW e *pqsE* q RV, using the Power SYBR GREEN PCR Master Mix (Applied Biosciences). The *oprL* gene was used as the internal control.

**Supporting Information**

**Figure S1:** RT-PCR analysis of the *pqsABCDE* operon. (A) Amplification of the individual *pqs* genes in PAO1. cDNA was synthesized from RNA extracted from *P. aeruginosa* PAO1 at various stages of growth (as determined by OD_600_). The corresponding RNA was amplified in parallel as a negative control, and the *P. aeruginosa* PAO1 genomic DNA was used as a positive control. (B) Amplification of the *pqsE* gene in PAO1 *pqsA* and PAO1 *pqsA pqsH* mutants. cDNA was synthesized from RNA extracted from cells grown to OD_600_ 1.5. Where indicated (+), strains were grown in presence of 40 μM HHQ and/or PQS. The corresponding RNA was amplified in parallel as a negative control, and the *P. aeruginosa* PAO1 genomic DNA was used as a positive control.


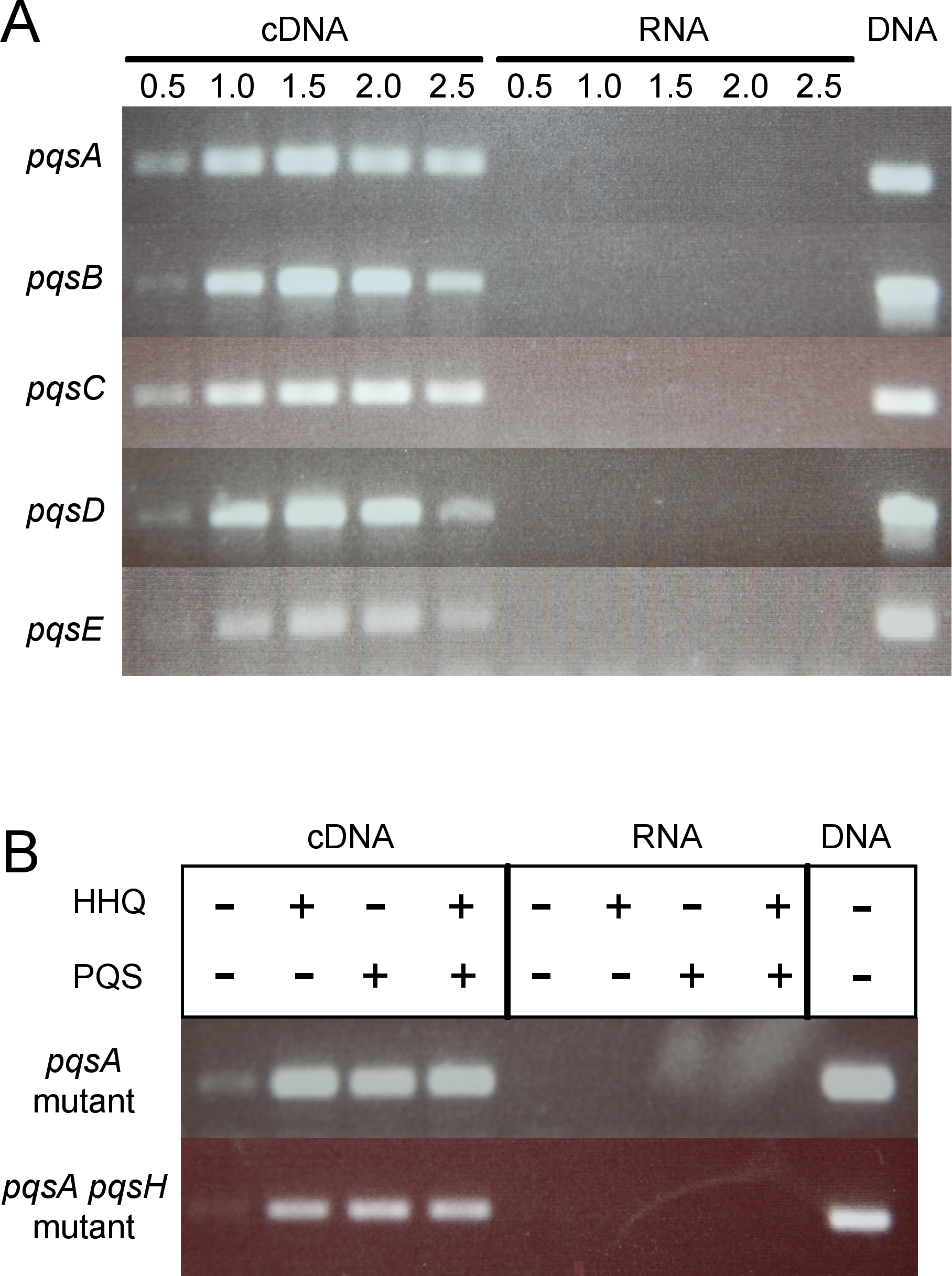


**Supporting Information**

**Figure S2:** Analysis of *pqsE* expression as a function of growth in the *pqsE*ind strain. The fold change in *pqsE* transcript levels in the pqsEind strain grown to early exponential phase (OD_600_ = 0.5) and late exponential phase (OD_600_ = 1.5) was determined by qRT-PCR in the presence of absence of IPTG. The relative expression of *pqsE* is compared with that of the wild type at an OD_600_ of 0.5. Where indicated, IPTG (1 mM) was added to the growth medium.


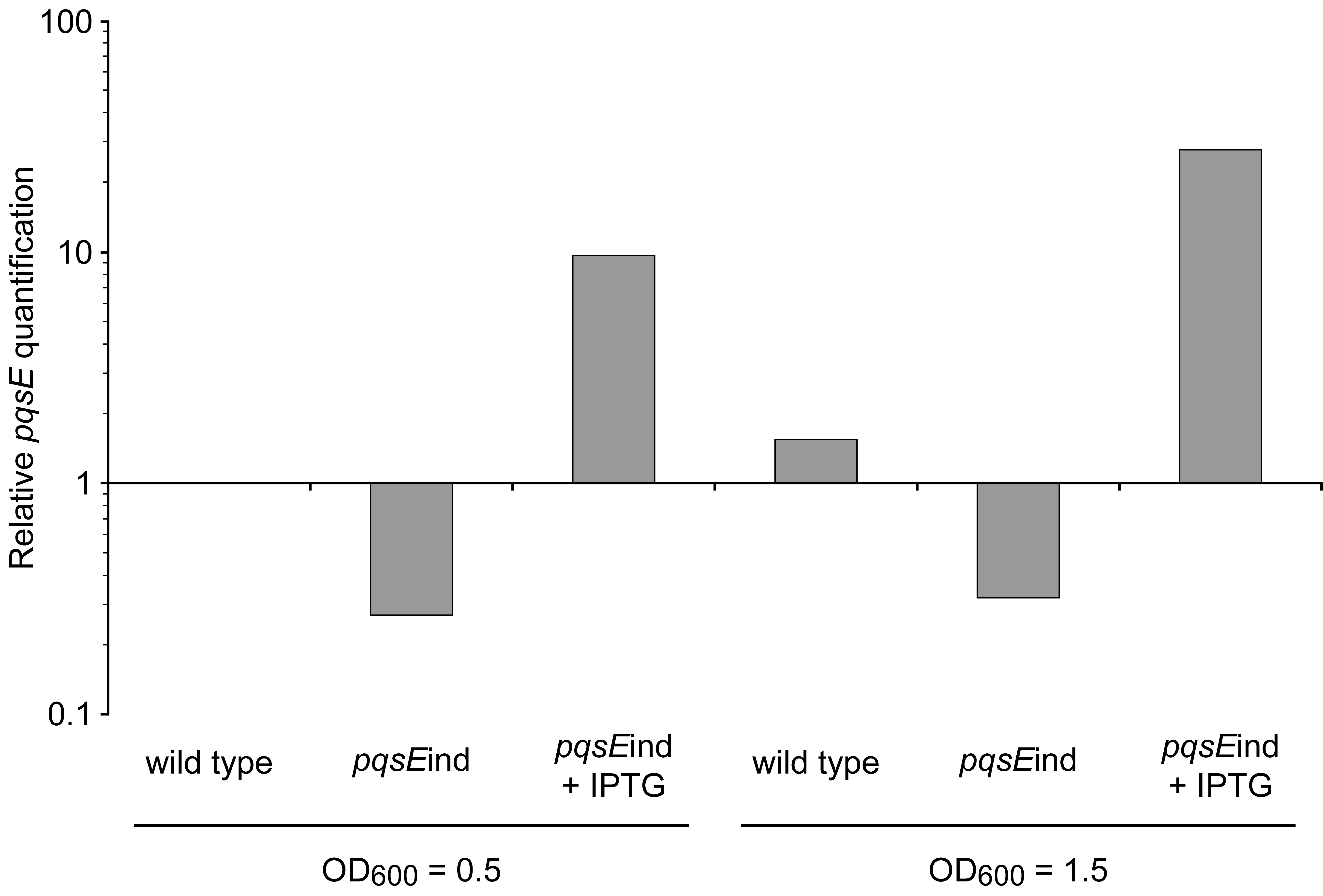


**Supporting Information**

**Table S1.** Genes regulated in the microarray experiments.

| **PA number ^a^** | **Gene name ^a^** | **wt vs *pqsA ^b^*** | **wt vs *pqsE*ind ^C^** | **wt vs *pqsE*ind+IPTG ^d^** | **Product name ^a^** |
| --- | --- | --- | --- | --- | --- |
| PA0041 | *-* |  |  | 1.599 | probable hemagglutinin |
| PA0047 | *-* |  |  | 1.519 | hypothetical protein |
| PA0051 | *phzH* |  |  | -1.597 | potential phenazine-modifying enzyme |
| **PA0052** | *-* |  |  | -1.546 | hypothetical protein |
| PA0060 | *-* |  |  | -1.545 | conserved hypothetical protein |
| PA0074 | *ppkA* |  | -1.761 |  | serine/threonine protein kinase PpkA |
| PA0090 | *clpV1* |  | -1.642 |  | ClpV1 |
| PA0126 | *-* |  |  | 1.778 | hypothetical protein |
| PA0140 ** | *ahpF* |  |  | -1.727 | alkyl hydroperoxide reductase subunit F |
| PA0142 | *-* |  |  | 1.538 | hypothetical protein |
| PA0146 | *-* |  |  | -1.677 | conserved hypothetical protein |
| PA0170 | *-* | 1.561 |  |  | hypothetical protein |
| PA0187 | *-* | 2.124 |  | -1.901 | hypothetical protein |
| PA0188 | *-* | 1.732 |  | -2.358 | hypothetical protein |
| PA0212 | *mdcE* | -3.288 |  |  | malonate decarboxylase gamma subunit |
| PA0226 | *-* | -7.437 |  | -12.590 | probable CoA transferase, subunit A |
| PA0227 | *pcaJ* |  |  | -50.240 | probable CoA transferase, subunit B |
| PA0228 | *pcaF* | -3.241 |  |  | beta-ketoadipyl CoA thiolase PcaF |
| PA0230 | *pcaB* |  |  | -2.909 | 3-carboxy-cis,cis-muconate cycloisomerase |
| PA0236 | *-* | 1.538 |  |  | probable transcriptional regulator |
| PA0243 | *-* |  |  | -1.950 | probable transcriptional regulator |
| PA0268 | *-* |  |  | 1.551 | probable transcriptional regulator |
| PA0269 | *-* | 1.684 |  | -1.861 | conserved hypothetical protein |
| PA0270 | *-* |  |  | -1.531 | hypothetical protein |
| PA0271 | *-* | 1.801 |  | -2.015 | hypothetical protein |
| PA0277 | *-* |  | -1.544 |  | conserved hypothetical protein |
| PA0295 | *-* |  |  | 1.713 | probable periplasmic polyamine binding protein |
| PA0347 | *glpQ* |  |  | 1.657 | glycerophosphoryl diester phosphodiesterase, periplasmic |
| PA0361 | *-* |  |  | 1.634 | probable gamma-glutamyltranspeptidase precursor |
| PA0363 | *coaD* |  |  | 1.663 | phosphopantetheine adenylyltransferase |
| **PA0365** | *-* | 1.573 |  |  | hypothetical protein |
| PA0386 | *yggW* | -2.540 |  |  | probable oxidase |
| PA0390 | *metX* | -1.510 |  |  | homoserine O-acetyltransferase |
| PA0420 | *bioA* |  |  | 1.661 | adenosylmethionine-8-amino-7-oxononanoate aminotransferase |
| **PA0435** | *-* |  |  | 2.239 | hypothetical protein |
| PA0438 | *codB* |  |  | 1.917 | cytosine permease |
| PA0444 | *hyuC* | 1.802 |  |  | N-carbamoyl-beta-alanine amidohydrolase |
| PA0452 | *slp* |  |  | -2.165 | probable stomatin-like protein |
| PA0471 | *fiuR* |  | -2.036 |  | probable transmembrane sensor |
| PA0477 | *-* |  |  | -1.803 | probable transcriptional regulator |
| PA0480 | *-* | 1.665 |  | -2.186 | probable hydrolase |
| PA0486 | *yihE* |  |  | 1.515 | conserved hypothetical protein |
| PA0545 | *-* | -3.333 | -1.979 |  | hypothetical protein |
| PA0630 | *-* |  |  | -1.591 | hypothetical protein |
| PA0733 | *rsuA* | -1.734 |  |  | probable pseudouridylate synthase |
| PA0751 | *-* |  |  | 1.988 | conserved hypothetical protein |
| PA0777 | *-* | -2.291 |  |  | hypothetical protein |
| PA0778 | *icp* |  |  | 1.505 | inhibitor of cysteine peptidase |
| PA0801 | *-* |  |  | 1.538 | hypothetical protein |
| PA0802 | *-* |  |  | 2.082 | hypothetical protein |
| PA0834 | *-* | -1.660 |  |  | conserved hypothetical protein |
| PA0860 | *-* |  |  | -1.502 | probable ATP-binding/permease fusion ABC transporter |
| PA0908 | *-* |  |  | -1.525 | hypothetical protein |
| **PA0996 *** | *pqsA* | 12.420 |  | 2.897 | probable coenzyme A ligase |
| **PA0997 * **** | *pqsB* | 7.350 |  | 3.801 | Homologous to beta-keto-acyl-acyl-carrier protein synthase |
| **PA0998 *** | *pqsC* | 6.597 |  | 3.735 | Homologous to beta-keto-acyl-acyl-carrier protein synthase |
| **PA0999 *** | *pqsD* | 6.466 |  | 2.804 | 3-oxoacyl-[acyl-carrier-protein] synthase III |
| **PA1000 *** | *pqsE* | 3.418 |  |  | Quinolone signal response protein |
| **PA1001 *** | *phnA* | 10.187 |  |  | anthranilate synthase component I |
| **PA1002 *** | *phnB* | 3.462 |  |  | anthranilate synthase component II |
| PA1100 | *fliE* |  |  | -1.570 | Flagellar hook-basal body complex protein FliE |
| PA1104 | *fliI* |  |  | -1.561 | Flagellum-specific ATP synthase FliI |
| PA1107 | *-* |  |  | -1.579 | conserved hypothetical protein |
| PA1141 | *-* |  |  | 1.553 | Probabile transcriptional regulator |
| PA1149 | *-* |  |  | 1.597 | hypothetical protein |
| PA1157 | *-* |  |  | 2.214 | Probabile two-component response regulator |
| PA1183 | *dctA* | -2.986 |  |  | C4-dicarboxylate transport protein |
| **PA1196** | *-* | -1.516 |  |  | Probabile transcriptional regulator |
| PA1197 | *-* | -2.485 |  |  | hypothetical protein |
| PA1211 | *-* | 1.648 |  | -2.186 | hypothetical protein |
| **PA1212** | *-* |  | 1.896 | -2.681 | probable MFS transporter |
| PA1213 | *-* | 2.274 | 1.669 |  | hypothetical protein |
| **PA1215** | *-* |  | 2.174 |  | hypothetical protein |
| **PA1218** | *-* | 2.458 |  |  | hypothetical protein |
| PA1220 | *-* | 3.373 | 1.913 | -2.704 | hypothetical protein |
| **PA1221** | *-* | 5.112 | 2.474 | -2.280 | hypothetical protein |
| PA1224 | *-* |  |  | 1.503 | Probabile NAD(P)H dehydrogenase |
| PA1233 | *-* |  |  | 2.235 | hypothetical protein |
| PA1242 | *-* | 1.856 | 1.749 |  | hypothetical protein |
| **PA1245 **** | *aprX* | 1.515 |  |  | hypothetical protein |
| **PA1246** | *aprD* | 1.624 |  |  | alkaline protease secretion protein AprD |
| PA1290 | *-* |  |  | 1.761 | probable transcriptional regulator |
| PA1301 | *-* |  |  | 2.014 | probable transmembrane sensor |
| **PA1317** | *cyoA* | 2.258 |  |  | cytochrome o ubiquinol oxidase subunit II |
| **PA1318** | *cyoB* | 2.027 |  |  | cytochrome o ubiquinol oxidase subunit I |
| **PA1319** | *cyoC* | 2.604 |  |  | cytochrome o ubiquinol oxidase subunit III |
| **PA1320** | *cyoD* | 2.817 |  |  | cytochrome o ubiquinol oxidase subunit IV |
| PA1331 | *yegH* |  | -1.545 |  | conserved hypothetical protein |
| PA1336 | *-* |  |  | -1.544 | probable two-component sensor |
| PA1407 | *-* |  |  | -1.522 | hypothetical protein |
| PA1411 | *-* |  |  | 1.613 | hypothetical protein |
| PA1429 | *-* | -1.702 |  |  | probable cation-transporting P-type ATPase |
| PA1452 | *flhA* |  |  | -1.509 | flagellar biosynthesis protein FlhA |
| PA1475 | *ccmA* |  |  | 1.721 | heme exporter protein CcmA |
| PA1506 | *-* | -1.619 |  |  | hypothetical protein |
| PA1519 | *-* |  |  | 1.616 | probable transporter |
| PA1540 | *-* |  |  | 1.565 | conserved hypothetical protein |
| PA1549 | *fixI* |  |  | -1.978 | probable cation-transporting P-type ATPase |
| **PA1559** | *-* |  |  | -1.581 | hypothetical protein |
| PA1603 | *-* | -1.590 |  |  | probable transcriptional regulator |
| PA1635 | *kdpC* |  | 1.937 |  | potassium-transporting ATPase, C chain |
| PA1638 | *yneH* | -1.533 |  |  | conserved hypothetical protein |
| PA1645 | *-* |  |  | 1.731 | hypothetical protein |
| PA1646 | *-* | 2.005 |  |  | probable chemotaxis transducer |
| PA1680 | *-* |  |  | -1.551 | hypothetical protein |
| PA1699 | *pcr1* |  |  | -1.596 | conserved hypothetical protein in type III secretion |
| PA1720 | *pscG* |  |  | -1.839 | type III export protein PscG |
| PA1722 | *pscI* |  |  | 1.590 | type III export protein PscI |
| PA1760 | *-* |  |  | -1.546 | probable transcriptional regulator |
| PA1765 | *-* | 1.537 |  |  | hypothetical protein |
| PA1783 | *nasA* |  |  | -1.841 | nitrate transporter |
| PA1837 | *-* | 1.862 |  |  | hypothetical protein |
| PA1839 | *-* | -1.600 |  |  | hypothetical protein |
| PA1872 | *-* |  |  | -1.537 | hypothetical protein |
| **PA1877** | *-* |  |  | -1.935 | probable secretion protein |
| PA1899 | *phzA2* | 2.556 |  |  | probable phenazine biosynthesis protein |
| **PA1901** | *phzC2* | 2.233 |  |  | phenazine biosynthesis protein PhzC |
| **PA1903** | *phzE2* | 2.016 |  |  | phenazine biosynthesis protein PhzE |
| PA1906 | *-* | 1.707 |  |  | hypothetical protein |
| PA1907 | *-* | 1.710 |  |  | hypothetical protein |
| **PA1921** | *-* | 2.127 |  |  | hypothetical protein |
| **PA1927** | *metE* |  |  | -12.014 | 5-methyltetrahydropteroyltriglutamate-homocysteine S-methyltransferase |
| PA1928 | *rimJ* | 1.560 |  |  | ribosomal protein alanine acetyltransferase |
| PA1931 | *-* |  |  | -1.768 | probable ferredoxin |
| PA1933 | *yagR* |  |  | -1.506 | probable hydroxylase large subunit |
| PA1964 | *ybiT* | -1.710 |  |  | probable ATP-binding component of ABC transporter |
| PA1982 | *exaA* |  |  | -1.512 | quinoprotein alcohol dehydrogenase |
| PA1990 | *-* |  |  | -1.681 | probable peptidase |
| PA2019 | *amrA* | -1.607 |  | 2.278 | RND, multidrug efflux membrane fusion protein precursor |
| PA2033 | *-* | 1.707 |  |  | hypothetical protein |
| PA2047 | *-* |  |  | -1.866 | probable transcriptional regulator |
| PA2048 | *-* |  | 1.505 |  | hypothetical protein |
| PA2062 | *-* |  | 1.668 |  | probable pyridoxal-phosphate dependent enzyme |
| **PA2066** | *-* | 2.230 |  | -1.919 | hypothetical protein |
| **PA2067 *** | *-* |  | 1.543 | -2.229 | probable hydrolase |
| **PA2068** | *-* | 2.951 | 1.682 |  | probable MFS transporter |
| **PA2069 *** | *-* | 2.161 |  |  | probable carbamoyl transferase |
| PA2089 | *-* |  |  | 1.559 | hypothetical protein |
| PA2107 | *-* | 1.564 |  |  | hypothetical protein |
| PA2115 | *-* |  |  | 2.825 | probable transcriptional regulator |
| PA2121 | *-* |  |  | -1.702 | probable transcriptional regulator |
| PA2126 ** | *-* | -2.869 |  |  | conserved hypothetical protein |
| PA2128 | *cupA1* | -2.062 | -1.581 |  | fimbrial subunit CupA1 |
| PA2140 | *-* |  |  | -2.237 | probable metallothionein |
| **PA2142** | *yhxC* |  |  | -1.698 | probable short-chain dehydrogenase |
| PA2145 | *-* | 1.582 |  |  | hypothetical protein |
| **PA2147** | *katE* |  |  | -1.925 | catalase HPII |
| **PA2148** | *-* |  |  | -1.676 | conserved hypothetical protein |
| PA2150 | *-* |  |  | -1.549 | conserved hypothetical protein |
| **PA2151** | *-* |  |  | -1.526 | conserved hypothetical protein |
| **PA2152** | *-* |  |  | -1.502 | probable trehalose synthase |
| **PA2153** | *glgB* |  | 1.641 |  | 1,4-alpha-glucan branching enzyme |
| PA2154 | *ybhN* |  |  | -2.102 | conserved hypothetical protein |
| PA2155 | *ybhO* | 1.557 |  |  | probable phospholipase |
| PA2162 | *-* |  |  | -2.329 | probable glycosyl hydrolase |
| **PA2163** | *-* |  |  | -2.298 | hypothetical protein |
| **PA2167** | *-* |  | 1.584 | -2.159 | hypothetical protein |
| **PA2168** | *-* |  |  | -2.628 | hypothetical protein |
| **PA2169** | *-* | 1.747 |  |  | hypothetical protein |
| **PA2170** | *-* |  |  | -1.620 | hypothetical protein |
| PA2179 | *-* |  |  | -1.690 | hypothetical protein |
| PA2181 | *-* |  |  | -1.741 | hypothetical protein |
| PA2183 | *-* | 1.505 |  |  | hypothetical protein |
| PA2184 | *yciE* | 1.984 | 1.812 |  | conserved hypothetical protein |
| **PA2192** | *-* | 1.560 |  |  | conserved hypothetical protein |
| PA2252 | *-* |  |  | 1.765 | probable AGCS sodium/alanine/glycine symporter |
| PA2273 | *soxR* | 2.947 | 2.099 |  | probable transcriptional regulator |
| **PA2274 * **** | *-* | 17.122 | 3.987 |  | hypothetical protein |
| PA2275 | *yahK* | 4.106 | 3.447 |  | probable alcohol dehydrogenase (Zn-dependent) |
| PA2287 | *-* | -1.837 |  |  | hypothetical protein |
| PA2289 | *-* |  |  | -1.827 | conserved hypothetical protein |
| PA2290 | *gcd* |  |  | -1.631 | glucose dehydrogenase |
| PA2297 | *-* |  |  | -1.706 | probable ferredoxin |
| PA2298 | *-* |  |  | -2.024 | probable oxidoreductase |
| PA2299 * | *-* |  |  | -2.023 | probable transcriptional regulator |
| **PA2300 *** | *chiC* | 1.707 |  |  | Chitinase |
| **PA2340** | *mtlG* |  |  | 1.543 | probable binding-protein-dependent maltose/mannitol transport protein |
| PA2375 | *-* |  |  | -1.588 | hypothetical protein |
| PA2407 | *-* |  | -2.158 |  | probable adhesion protein |
| PA2409 | *-* |  | -2.053 |  | probable permease of ABC transporter |
| PA2412 ** | *-* | 1.786 |  |  | conserved hypothetical protein |
| PA2413 | *pvdH* | 1.674 |  |  | L-2,4-diaminobutyrate:2-ketoglutarate 4-aminotransferase |
| **PA2414** | *-* | 2.189 |  |  | L-sorbosone dehydrogenase |
| **PA2415** | *-* | 1.791 |  |  | hypothetical protein |
| PA2416 | *treA* | 2.655 |  |  | periplasmic trehalase precursor |
| PA2420 | *opdJ* |  |  | 2.013 | probable porin |
| PA2430 | *-* |  |  | -1.826 | conserved hypothetical protein |
| PA2431 | *-* | 1.595 |  |  | hypothetical protein |
| **PA2448** | *-* |  |  | -2.449 | hypothetical protein |
| PA2462 | *-* |  |  | 1.502 | hypothetical protein |
| PA2467 | *foxR* |  |  | 1.615 | Anti-sigma factor FoxR |
| PA2480 | *-* |  |  | 1.606 | probable two-component sensor |
| PA2506 | *-* | -2.349 |  | -4.105 | hypothetical protein |
| PA2507 | *catA* | -3.125 |  | -5.165 | catechol 1,2-dioxygenase |
| PA2508 | *catC* | -2.544 | 4.185 | -3.756 | muconolactone delta-isomerase |
| PA2509 | *catB* | -3.597 | 3.577 | -6.172 | muconate cycloisomerase I |
| PA2511 | *-* |  | 3.027 | -1.791 | probable transcriptional regulator |
| PA2519 | *xylS* | -2.077 |  |  | transcriptional regulator XylS |
| **PA2570 *** | *lecA* | 2.219 |  |  | LecA |
| PA2589 | *-* |  |  | -1.500 | hypothetical protein |
| PA2606 | *yheM* |  |  | -1.601 | conserved hypothetical protein |
| PA2608 | *yccK* |  |  | -1.523 | conserved hypothetical protein |
| PA2665 | *ygaA* |  |  | 1.893 | probable transcriptional regulator |
| PA2666 | *ptpS* | -1.787 |  |  | probable 6-pyruvoyl tetrahydrobiopterin synthase |
| PA2679 | *-* |  |  | -1.600 | hypothetical protein |
| PA2682 | *-* |  |  | -1.668 | conserved hypothetical protein |
| PA2702 | *-* |  | -1.602 |  | hypothetical protein |
| PA2742 | *rpmI* |  |  | 1.512 | 50S ribosomal protein L35 |
| PA2787 | *cpg2* |  |  | -1.623 | carboxypeptidase G2 precursor |
| PA2788 | *-* |  |  | -1.814 | probable chemotaxis transducer |
| PA2810 | *copS* |  |  | 1.667 | two-component sensor, CopS |
| PA2829 | *-* |  |  | 1.693 | hypothetical protein |
| PA2857 | *-* |  |  | 1.805 | probable ATP-binding component of ABC transporter |
| PA2868 ** | *-* |  |  | -1.757 | hypothetical protein |
| PA2871 | *-* | 1.569 |  |  | hypothetical protein |
| PA2876 | *pyrF* |  |  | 1.558 | orotidine 5'-phosphate decarboxylase |
| PA2888 | *atuC* |  | 1.502 | -1.589 | geranyl-CoA carboxylase, beta-subunit |
| PA2892 | *atuG* |  |  | -1.515 | GCase, alpha-subunit (biotin-containing) |
| PA2898 | *-* |  |  | -1.548 | hypothetical protein |
| PA2906 | *-* | -1.546 |  |  | probable oxidoreductase |
| PA2912 | *-* |  |  | -1.611 | probable ATP-binding component of ABC transporter |
| PA2920 | *-* |  |  | -1.707 | probable chemotaxis transducer |
| **PA2927** | *-* |  |  | -1.725 | hypothetical protein |
| PA2928 | *-* |  |  | -1.572 | hypothetical protein |
| PA2956 | *-* |  |  | 1.500 | conserved hypothetical protein |
| PA3023 | *yegS* |  | 1.509 |  | conserved hypothetical protein |
| PA3037 | *-* | 1.533 |  |  | hypothetical protein |
| PA3039 | *-* | 1.666 |  |  | probable transporter |
| PA3089 | *-* |  |  | -2.238 | hypothetical protein |
| PA3095 | *xcpZ* |  |  | -1.895 | general secretion pathway protein M |
| PA3096 | *xcpY* |  |  | -1.620 | general secretion pathway protein L |
| PA3098 | *xcpW* |  |  | -1.813 | general secretion pathway protein J |
| PA3099 | *xcpV* |  |  | -1.948 | general secretion pathway protein I |
| PA3102 | *xcpS* |  |  | -1.520 | general secretion pathway protein F |
| PA3132 | *-* | 1.860 |  |  | probable hydrolase |
| **PA3182** | *pgl* |  | -1.731 |  | 6-phosphogluconolactonase |
| PA3187 | *gltK* |  |  | 2.456 | probable ATP-binding component of ABC transporter |
| **PA3189 *** | *gltF* |  |  | 2.123 | probable permease of ABC sugar transporter |
| PA3208 | *ydjA* |  |  | 1.582 | conserved hypothetical protein |
| PA3213 | *-* |  |  | -1.530 | hypothetical protein |
| PA3221 | *csaA* | -1.791 |  |  | CsaA protein |
| PA3269 | *-* |  |  | 1.537 | probable transcriptional regulator |
| PA3270 | *-* |  |  | 1.542 | hypothetical protein |
| **PA3284** | *-* | 1.673 |  |  | hypothetical protein |
| PA3287 ** | *-* |  |  | -2.179 | conserved hypothetical protein |
| PA3289 | *-* |  |  | -1.531 | hypothetical protein |
| PA3296 | *phoA* | -2.046 | -1.724 |  | alkaline phosphatase |
| PA3304 | *-* |  |  | -1.684 | conserved hypothetical protein |
| PA3309 | *uspK* | -1.659 |  |  | conserved hypothetical protein |
| PA3314 | *-* |  |  | 2.948 | probable ATP-binding component of ABC transporter |
| **PA3327** | *-* |  |  | 3.756 | probable non-ribosomal peptide synthetase |
| **PA3328** | *-* |  |  | 5.081 | probable FAD-dependent monooxygenase |
| **PA3336** | *-* |  |  | 1.657 | probable MFS transporter |
| PA3372 | *phnP* | 1.722 |  |  | conserved hypothetical protein |
| PA3388 | *yaeB* | -1.625 |  |  | conserved hypothetical protein |
| PA3417 | *-* |  |  | -1.670 | probable pyruvate dehydrogenase E1 component, alpha subunit |
| PA3419 | *-* | 1.526 |  |  | hypothetical protein |
| PA3460 | *-* |  |  | -1.512 | probable acetyltransferase |
| PA3470 | *-* |  |  | 1.600 | hypothetical protein |
| PA3493 | *rnfG* | 2.066 |  | -1.656 | conserved hypothetical protein |
| **PA3520 * **** | *-* | 1.507 |  |  | hypothetical protein |
| PA3553 | *arnC* |  |  | 1.681 | ArnC |
| PA3556 | *arnT* |  |  | 1.644 | inner membrane L-Ara4N transferase ArnT |
| PA3572 | *-* |  | -1.604 |  | hypothetical protein |
| PA3573 | *-* |  |  | 2.304 | probable MFS transporter |
| PA3578 | *-* | 1.748 | 1.649 |  | conserved hypothetical protein |
| PA3581 | *glpF* | -3.131 |  | -2.310 | glycerol uptake facilitator protein |
| PA3596 | *-* | 1.994 |  |  | probable methylated-DNA--protein-cysteine methyltransferase |
| PA3607 | *potA* |  |  | -1.566 | polyamine transport protein PotA |
| PA3633 | *ygbP* | -2.108 |  |  | 4-diphosphocytidyl-2-C-methylerythritol synthase |
| PA3665 | *-* |  |  | 1.568 | hypothetical protein |
| PA3667 | *-* |  |  | -1.573 | probable pyridoxal-phosphate dependent enzyme |
| PA3671 | *-* |  |  | 2.099 | probable permease of ABC transporter |
| PA3672 | *-* |  |  | 2.582 | probable ATP-binding component of ABC transporter |
| **PA3676** | *-* | 1.655 |  | 2.043 | RND, efflux transporter |
| **PA3677** | *-* | 1.909 |  | 4.780 | RND, efflux membrane fusion protein precursor |
| **PA3678 *** | *-* |  |  | 1.572 | probable transcriptional regulator |
| PA3679 | *-* |  |  | 1.691 | hypothetical protein |
| PA3718 | *-* | 2.242 |  |  | probable MFS transporter |
| PA3840 | *ybiN* | -1.710 |  | -1.521 | conserved hypothetical protein |
| **PA3875** | *narG* | -2.552 |  |  | respiratory nitrate reductase alpha chain |
| PA3879 | *narL* | -2.216 |  |  | two-component response regulator NarL |
| PA3886 | *-* |  |  | 1.684 | hypothetical protein |
| PA3894 | *opmI* |  |  | 2.198 | probable outer membrane protein precursor |
| PA3901 | *fecA* |  | -1.668 |  | Fe(III) dicitrate transport protein FecA |
| PA3909 | *-* |  |  | -1.867 | hypothetical protein |
| PA3925 | *-* |  |  | 2.170 | probable acyl-CoA thiolase |
| PA3959 | *-* | -1.811 |  |  | hypothetical protein |
| PA3968 | *ymfC* |  |  | 2.416 | probable pseudouridine synthase |
| PA3983 | *-* |  |  | -1.851 | conserved hypothetical protein |
| PA4017 | *yraR* |  |  | -1.953 | conserved hypothetical protein |
| PA4027 | *-* |  |  | -1.585 | hypothetical protein |
| PA4041 | *-* | 1.629 |  |  | hypothetical protein |
| PA4063 | *-* |  |  | -3.015 | hypothetical protein |
| PA4110 | *ampC* |  |  | 1.687 | beta-lactamase precursor |
| PA4112 | *-* |  | 1.553 |  | probable sensor/response regulator hybrid |
| **PA4134** | *-* |  |  | -1.550 | hypothetical protein |
| PA4140 | *-* |  |  | -2.307 | hypothetical protein |
| **PA4141 * **** | *-* |  |  | -4.516 | hypothetical protein |
| **PA4142** | *-* | 1.650 |  | -6.713 | probable secretion protein |
| PA4143 | *cyaB* |  |  | -4.311 | probable toxin transporter |
| PA4144 | *opmK* |  |  | -4.160 | probable outer membrane protein precursor |
| PA4145 | *-* |  |  | -1.550 | probable transcriptional regulator |
| **PA4171** | *-* | 1.631 |  |  | probable protease |
| PA4195 | *-* |  | 1.790 |  | probable binding protein component of ABC transporter |
| **PA4205 * **** | *mexG* | 2.944 |  |  | hypothetical protein |
| **PA4206 * **** | *mexH* | 5.338 |  |  | RND, efflux membrane fusion protein precursor |
| **PA4207 *** | *mexI* | 7.925 | 1.886 |  | RND, efflux transporter |
| **PA4208 *** | *opmD* | 20.813 | 6.835 |  | probable outer membrane protein precursor |
| **PA4210** | *phzA1* | 2.440 | 1.519 |  | probable phenazine biosynthesis protein |
| **PA4212 *** | *phzC1* | 2.131 |  |  | phenazine biosynthesis protein PhzC |
| **PA4214 *** | *phzE1* | 1.678 |  | -1.629 | phenazine biosynthesis protein PhzE |
| PA4218 ** | *-* | 4.928 | 4.940 |  | probable transporter |
| PA4221 ** | *fptA* | 7.076 | 5.448 | 6.577 | Fe(III)-pyochelin outer membrane receptor precursor |
| PA4222 ** | *pchI* | 7.157 | 11.923 | 7.876 | probable ATP-binding component of ABC transporter |
| PA4225 ** | *pchF* | 71.483 | 57.723 | 75.785 | pyochelin synthetase |
| PA4226 ** | *pchE* | 139.217 | 94.197 | 124.376 | dihydroaeruginoic acid synthetase |
| PA4227 ** | *pchR* | 8.728 | 3.245 | 7.757 | Transcriptional regulator PchR |
| PA4228 ** | *pchD* | 21.066 | 16.892 | 20.643 | pyochelin biosynthesis protein PchD |
| PA4229 ** | *pchC* | 1.611 |  |  | pyochelin biosynthetic protein PchC |
| PA4230 ** | *pchB* | 27.171 | 15.570 |  | salicylate biosynthesis protein PchB |
| PA4231 ** | *pchA* | 32.538 | 21.940 | 38.831 | salicylate biosynthesis isochorismate synthase |
| PA4290 | *-* |  |  | -2.728 | probable chemotaxis transducer |
| PA4292 | *-* | -1.572 |  |  | probable phosphate transporter |
| **PA4293** | *pprA* |  |  | -1.585 | two-component sensor PprA |
| **PA4300** | *tadC* |  |  | -1.525 | TadC |
| **PA4302** | *tadA* | 1.738 |  |  | TadA ATPase |
| **PA4303** | *tadZ* | 1.946 |  |  | TadZ |
| **PA4305** | *rcpC* | 2.337 |  |  | RcpC |
| PA4344 | *-* |  | 1.685 |  | probable hydrolase |
| **PA4371** | *-* |  |  | 2.412 | hypothetical protein |
| **PA4384** | *-* | 1.606 |  | -1.974 | hypothetical protein |
| PA4438 | *yhcM* | -1.630 |  |  | conserved hypothetical protein |
| PA4469 ** | *-* | 1.512 |  |  | hypothetical protein |
| PA4470 ** | *fumC1* | 1.657 |  |  | fumarate hydratase |
| PA4471 ** | *fagA* | 1.598 |  |  | hypothetical protein |
| PA4485 | *-* | -1.935 |  |  | conserved hypothetical protein |
| PA4513 | *piuB* | 2.340 |  |  | probable oxidoreductase |
| PA4516 | *-* |  |  | 1.506 | hypothetical protein |
| PA4588 | *gdhA* | -1.518 |  |  | glutamate dehydrogenase |
| PA4597 | *oprJ* |  |  | 1.968 | Multidrug efflux outer membrane protein OprJ precursor |
| PA4601 | *morA* | -2.129 | -1.568 |  | motility regulator |
| PA4612 | *ankB* |  |  | -1.614 | conserved hypothetical protein |
| PA4613 ** | *katB* |  |  | -2.045 | Catalase |
| PA4622 | *-* |  |  | -1.504 | probable MFS transporter |
| PA4627 | *yjjT* | -2.024 |  |  | conserved hypothetical protein |
| PA4680 | *-* |  |  | -1.664 | hypothetical protein |
| PA4771 | *lldD* |  |  | 1.743 | L-lactate dehydrogenase |
| PA4783 | *yedA* |  |  | 1.587 | conserved hypothetical protein |
| PA4821 | *dinF* |  |  | 4.503 | probable transporter |
| PA4844 | *-* | -1.929 |  |  | probable chemotaxis transducer |
| PA4845 | *dipZ* |  |  | 1.604 | thiol:disulfide interchange protein DipZ |
| PA4855 | *purD* |  |  | 1.653 | phosphoribosylamine-glycine ligase |
| PA4889 | *-* | -2.409 |  |  | probable oxidoreductase |
| PA4890 | *desT* | -1.595 |  |  | DesT |
| **PA4918** | *-* |  |  | 1.767 | hypothetical protein |
| PA4944 | *hfq* |  |  | 1.742 | Hfq |
| PA4949 | *yjeF* |  |  | 1.528 | conserved hypothetical protein |
| PA4975 | *-* |  |  | 1.802 | NAD(P)H quinone oxidoreductase |
| PA4977 | *aruI* |  |  | 1.555 | 2-ketoarginine decarboxylase, AruI |
| PA4983 | *dmsR* |  |  | 1.712 | probable two-component response regulator |
| PA4986 | *-* |  |  | 1.897 | probable oxidoreductase |
| PA5023 | *ydiU* |  | 1.756 | -1.641 | conserved hypothetical protein |
| PA5071 | *-* | -1.873 |  |  | conserved hypothetical protein |
| PA5137 | *-* |  |  | 1.685 | hypothetical protein |
| PA5180 | *fdhD* |  |  | -1.563 | conserved hypothetical protein |
| PA5201 | *yhgF* |  |  | 1.910 | conserved hypothetical protein |
| PA5228 | *ygfA* | -1.647 |  |  | conserved hypothetical protein |
| **PA5231** | *yhiH* | -1.724 |  |  | probable ATP-binding/permease fusion ABC transporter |
| PA5252 | *yheS* |  |  | 1.556 | probable ATP-binding component of ABC transporter |
| PA5340 | *-* |  |  | -1.634 | hypothetical protein |
| **PA5352** | *glcG* |  | 1.591 |  | conserved hypothetical protein |
| PA5362 | *-* | -1.574 |  |  | conserved hypothetical protein |
| PA5366 | *pstB* |  |  | 1.559 | ATP-binding component of ABC phosphate transporter |
| PA5367 | *pstA* |  |  | 2.118 | membrane protein component of ABC phosphate transporter |
| PA5368 | *pstC* |  |  | 1.657 | membrane protein component of ABC phosphate transporter |
| PA5369 | *pstS* |  |  | 1.824 | phosphate ABC transporter, periplasmic phosphate-binding protein |
| PA5375 | *betT1* | 1.530 |  |  | choline transporter BetT |
| PA5395 | *-* | 1.504 |  |  | conserved hypothetical protein |
| PA5434 | *mtr* | -1.881 | -1.534 |  | tryptophan permease |
| PA5500 | *znuC* | -2.037 |  | -2.135 | zinc transport protein ZnuC |
| PA5522 | *-* |  | 1.513 |  | probable glutamine synthetase |

^a^Gene number, gene name and product name are from the Pseudomonas Genome Database ([http://www.pseudomonas.com](http://www.pseudomonas.com/)). Genes previously reported to be QS-controlled are in bold characters (Hentzer *et al*., 2003; Schuster *et al*., 2003; Wagner *et al*., 2003). Single asterisk (*) indicates genes regulated by PqsR (MvfR) in Déziel *et al*., (2005); double asterisk (**) indicates genes regulated by PQS in Bredenbruch *et al*., (2006); RND, resistance-nodulation-cell division; MFS, major facilitator superfamily.

^b^ Fold change in gene expression of *P. aeruginosa* PAO1 wild type (wt) compared with *P. aeruginosa* PAO1 *pqsA* mutant (*pqsA*).

^c^ Fold change in gene expression of *P. aeruginosa* PAO1 wild type (wt) compared with *P. aeruginosa* PAO1 *pqsE*ind strain (*pqsE*ind).

^d^ Fold change in gene expression of *P. aeruginosa* PAO1 wild type (wt) compared with *P. aeruginosa* PAO1 *pqsE*ind strain grown in presence of 1 mM IPTG (*pqsE*ind+IPTG).

**Supporting Information**

**Table S2.** Primers used in this work.

| Name | Sequence * | Restriction site |
| --- | --- | --- |
| *rhlR*Up1 | 5’-ATATCTAGATACGCGCCACTGGGAGCC-3’ | XbaI |
| *rhlR*Up2 | 5’-GCGAAGCTTTCCGTCATTCCTCATTGC-3’ | HindIII |
| *rhlR*Dw1 | 5’-ACAAAGCTTCTCATCTGAAGCGCAGGG-3’ | HindIII |
| *rhlR*Dw2 | 5’-ATAGTCGACCGAAAGCTCCCAGACCGA-3’ | SalI |
| *pqsE*Up1 | 5’-ATAAAGCTTGCCGGCGAGAGTCTCGAA-3’ | HindIII |
| *pqsE*Up2 | 5’-ATAGGATCCAAGCCTCAACATGGCCGGT-3’ | BamHI |
| *pqsE*Dw1 | 5’-ATAGGATCCCTGGACTGAGACGGGACAT-3’ | BamHI |
| *pqsE*Dw2 | 5’-ATATCTAGAAGGCTGGACAGGCCATGC-3’ | XbaI |
| EindFW1 | 5’-TGCTCTAGACGGCATGTCCTGGTGGTC-3’ | XbaI |
| EindRV1 | 5’-TATGGATCCGGAGCCGAAAGCCTCAACA-3’ | BamHI |
| EindFW2 | 5’-CCGGAATTCATGTTGAGGCTTTCGGCTC-3’ | EcoRI |
| EindRV2 | 5’-TCCGCTCGAGGACTCCAGGTAAGCCTCC-3’ | XhoI |
| *pqsA*FW | 5’-GCCGGACCTACATTCTCTC-3’ | - |
| *pqsA*RV | 5’-GCATCGGCTTCACGCACC-3’ | - |
| *pqsB*FW | 5’-CCGCTCGAGCGACCAGGGCTATCGCA-3’ | XhoI |
| *pqsB*RV | 5’-CCGGAATTCCTTATGCATGAGCTTCTCC-3’ | EcoRI |
| *pqsC*FW | 5’-GCTCGCAGCTACGAAAACG-3’ | - |
| *pqsC*RV | 5’-CGAGGAAGCCGAACAGATC-3’ | - |
| *pqsD*FW | 5’-CCGGAATTCTGCTGAGGCATCGCCATGT-3’ | EcoRI |
| *pqsD*RV | 5’-TGCTCTAGACCGAGCAGGATCGACAGG-3’ | XbaI |
| *pqsE*FW | 5’-CGGTGTTCCTGCTGCGTC-3’ | - |
| *pqsE*RV | 5’-GACGCCAGGACCTGTACG-3’ | - |
| *pqsE*18FW | 5’-CAGCGGGAGCTCGTCCTGACCTACGGCTCCGGCGCG-3’ | SacI |
| *pqsE*18RV | 5’-CGCCGCTCTAGATCAGTGACTGTGATGGTGATGGTCCAGAGGCAGCGCCTG-3’ | XbaI |
| *pqsE* q FW | 5’-GGGCGCTGGTTGAAGGA-3’ | - |
| *pqsE* q RV | 5’-GTCCGCCCAAACCAATTC-3’ | - |

* Restriction sites are underlined in the primer sequences.
